# Protein nanoparticles in drug delivery: animal protein, plant proteins and protein cages, albumin nanoparticles

**DOI:** 10.1186/s12951-021-00896-3

**Published:** 2021-05-29

**Authors:** Ehsan Kianfar

**Affiliations:** 1 ERNAM-Erciyes University Nanotechnology Application and Research Center, Kayseri, 38039 Turkey; 2 Department of Analytical Chemistry, Faculty of Pharmacy, Erciyes University, Kayseri, 38039 Turkey

**Keywords:** Protein nanoparticles, Drug delivery, Animal protein, Plant proteins, Protein cages, Albumin nanoparticles

## Abstract

In this article, we will describe the properties of albumin and its biological functions, types of sources that can be used to produce albumin nanoparticles, methods of producing albumin nanoparticles, its therapeutic applications and the importance of albumin nanoparticles in the production of pharmaceutical formulations. In view of the increasing use of Abraxane and its approval for use in the treatment of several types of cancer and during the final stages of clinical trials for other cancers, to evaluate it and compare its effectiveness with conventional non formulations of chemotherapy Paclitaxel is paid. In this article, we will examine the role and importance of animal proteins in Nano medicine and the various benefits of these biomolecules for the preparation of drug delivery carriers and the characteristics of plant protein Nano carriers and protein Nano cages and their potentials in diagnosis and treatment. Finally, the advantages and disadvantages of protein nanoparticles are mentioned, as well as the methods of production of albumin nanoparticles, its therapeutic applications and the importance of albumin nanoparticles in the production of pharmaceutical formulations.
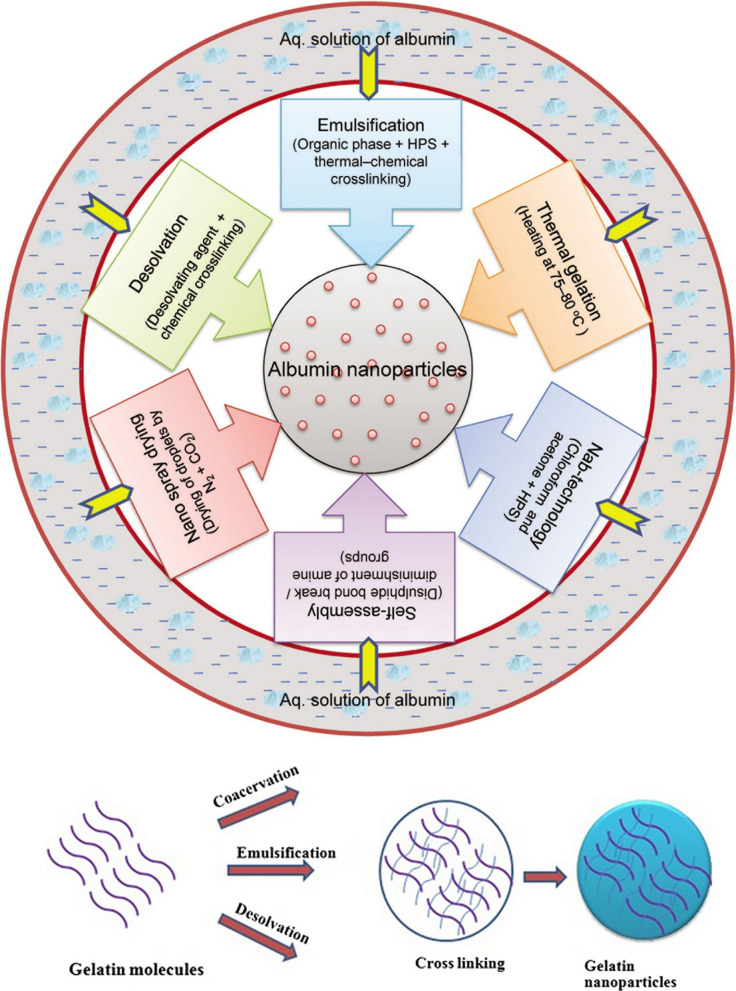

## Introduction

Proteins and peptides are one of the most important and basic research fields in Nano medicine. At present, experts in various fields in Nano medicine, Nano biotechnology, pharmacy, toxicology, immunology and other medical sciences are studying the various dimensions of these vital biomolecules, including understanding the interaction of the resulting nanostructures with the body and their application in diagnostic fields and are engaged in therapy [1–8]. Plants have been continuously considered by researchers as safe and abundant, renewable and cheap resources in various pharmaceutical, medical and food industries. Nano carriers made from some plant proteins have the ability to control the release of their cargo over a long period of time [9, 10]. The possibility of transmitting the disease from plant sources to humans is rare, and for this reason, the use of plant proteins to produce nano carriers for therapeutic agents has been considered by experts in recent years [11–17]. On the other hand, some protein subunits, such as the capsid proteins of viruses, have the ability to self-assemble and create hollow nanometer structures with well-defined and reproducible geometric shapes [18, 19]. The empty space inside these nanostructures or their surface can be used as a reservoir to carry pharmaceutical and diagnostic agents. These nanostructures are called protein cages and have great potential in preparing pharmaceutical formulations. Albumin is one of the animal proteins that has been considered since the early twentieth century and has several therapeutic applications [20–22]. On the one hand, this protein has therapeutic applications and on the other hand, it is used in various formulations to carry pharmacological and diagnostic agents [23–25]. The many benefits and potentials of this protein have made it the focus of Nano medical researchers [17, 26, 27]. Abraxane is a drug formulation based on albumin nanoparticles that sold more than 2 $ billion in 2012 alone and is considered by experts to be one of the main approaches to treating all types of cancer in the near future [28, 29]. The following section covers the most recent uses of therapeutic nanoparticles as selective delivery mechanisms in a variety of diseases, and Table 1 summarizes the nano-drug formulations authorised by the Food and Drug Administration (FDA) and the Euro.

**Table 1:** Food and Drug Administration and European Medicines Agency approved therapeutic nanoparticles

Nanostructure	Production	Nanoparticle formulation	Drug	Indication(s)	Confirmed	Refs.
Liposomes	Marqibo^®^	Sphingomyelin and cholesterol	Vincristine sulfate	Acute lymphoid leukemia	FDA 2012	[30]
Liposomes	Mepact^®^	1-Palmitoyl-2-oleoyl-snglycero-3-phosphocholine and 1,2-Dioleoyl-sn-glycero-3-phospho-l-serine liposomes	Mifamurtide	Non-metastasizing osteosarcoma	Europe 2009	[31]
Liposomes	Onivyde^®^	Nanoliposomes	Irinotecan	Pancreatic cancer, colorectal cancer	FDA 2015 Europe 2016	[32]
Liposomes	Vyxeos^®^	Distearoylphosphatidylcholine, distearoylphosphatidylglycerol, cholesterol	Daunorubicin Cytarabine	Acute myeloid leukemia	FDA 2017	[33]
Lipid-based (non-liposoma)	Onpattro^®^	Lipid nanoparticles	Transthyretin targeted siRNA	Transthyretin-mediated amyloidosis	FDA 2018	[34]
Polymer-based	Glatopa^®^	l-glutamic acid polymer with l-alanine, l-lysine, and l-tyrosine (Glatiramer)	–-	Multiple sclerosis	FDA 2015	[35]
Protein-drug conjugates	Kadcyla^®^	Maytansine derivative, DM1	Trastuzumab	HER2 + breast cancer	FDA 2013	[36]
Protein-drug conjugates	Abraxane^®^	Albumin	Paclitaxel	Non-small lung cancer, pancreatic cancer	FDA 2012 Europe 2005, FDA 2013 Europe 2008	[37]
Protein-drug conjugates	Krystexxa^®^	PEGylated uricase	Pegloticase	Gout disease	FDA 2010 Europe 2013	[38]
Protein-drug conjugates	Plegridy^®^	PEGylated interferon-1a	Interferon-1a	Multiple sclerosis	FDA 2014 Europe 2014	[38]
Protein-drug conjugates	Adynovate^®^	PEGylated factor VIII	Factor VIII	Hemophilia	FDA 2015	[39]
Protein-drug conjugates	Rebinyn^®^	Glycopegylated coagulation factor IX	Factor IX	Hemophilia	FDA 2015	[40]

Pean Medicines Agency (EMA) since 2009

## Nano medicine and proteins

### Nano medicine and proteins in the field of treatment

**Fig. 1:**
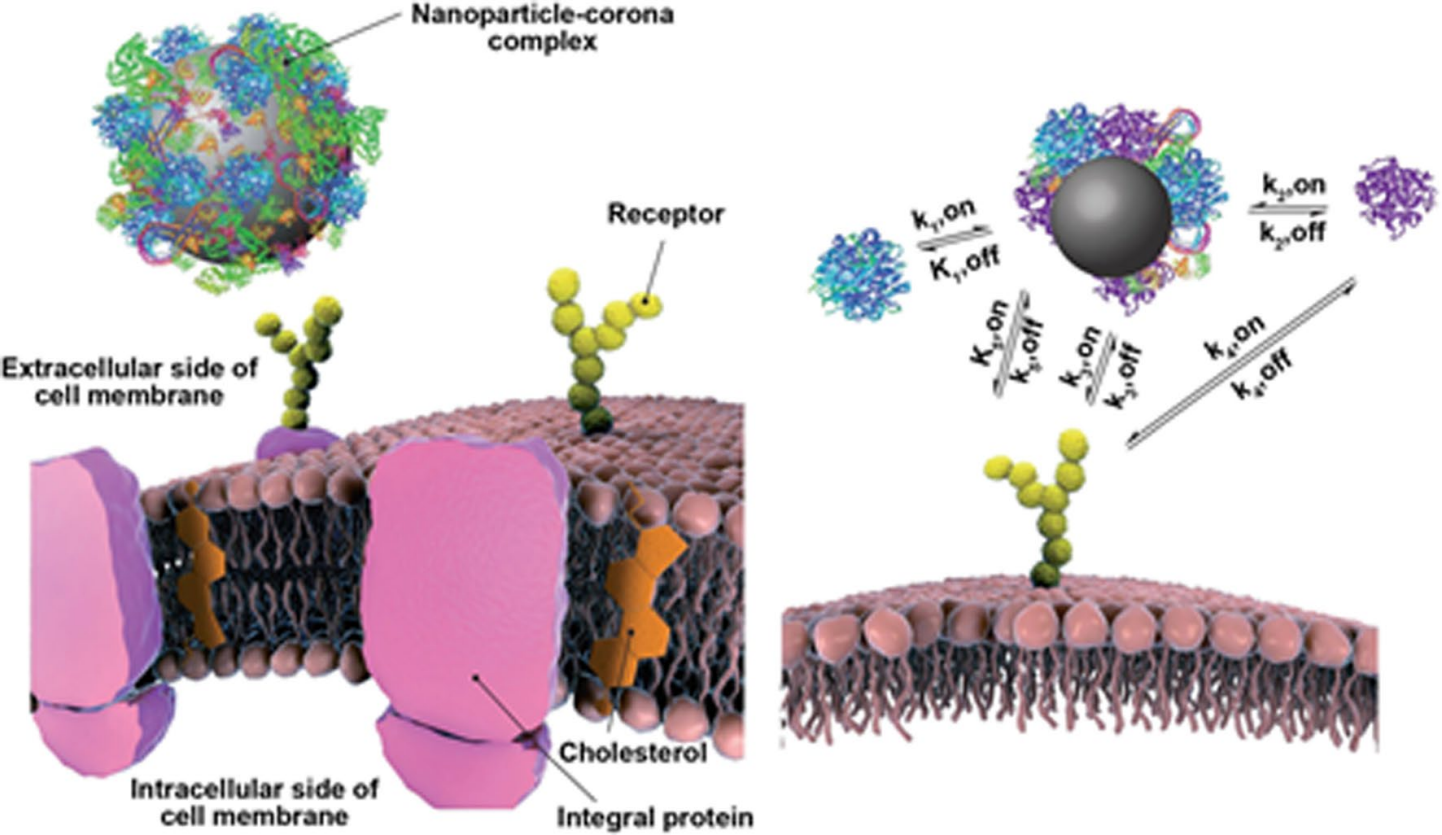
Nanoparticle protein crown: the formation of a protein crown around transferrin-targeted nanoparticles obscures the second transferrin binding and prevents it from binding to the surface of the target cell [48–51]

**Fig. 2:**
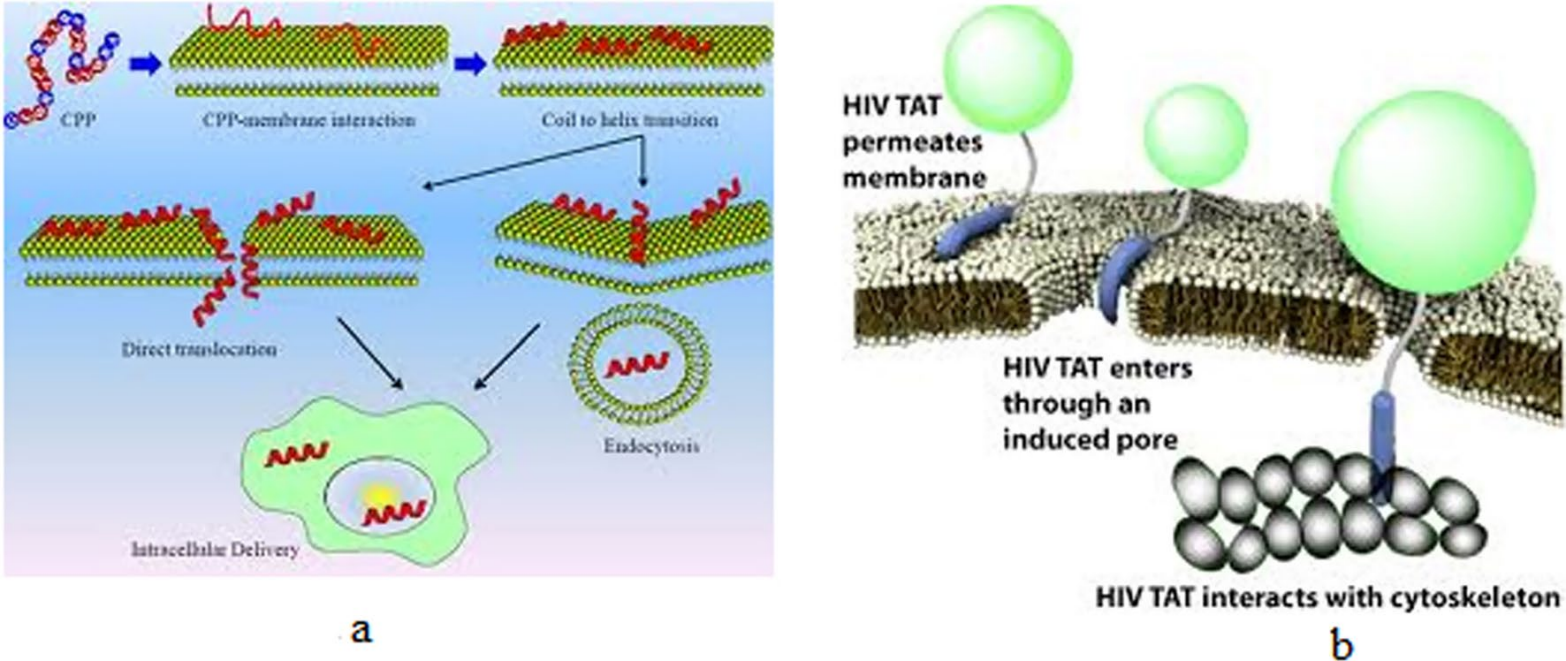
Cell penetrating peptides: **a** cell-penetrating peptides enter the cell by various mechanisms directly or through endocytosis pathways and can enter the cell-bound cargo. **b** AIDS TAT peptide is one of the cell-penetrating peptides that has been used in various studies to introduce nanoparticles into cells [48–51]

Many proteins and peptides such as insulin, vaccines, antibodies and various recombinant proteins have been used in medicine and therapy, and among the important research areas in Nano medicine is the development of new drug delivery systems to improve their function and properties [41–43]. On the other hand, one of the most important challenges facing Nano pharmaceutical formulations is the interaction of different blood proteins with them and the formation of a protein crown (Protein corona) around the nanoparticles, which plays an important role in the final performance of nanoparticles (Fig. 1). These include immune system proteins, including antibodies, and complement systems. Extensive efforts are being made to control the interaction of drug formulations with a variety of proteins, especially immune system proteins, to improve the performance of Nano drugs [44–47]. Various peptides and proteins have also been considered to target drug-containing nanoparticles to target tissue or tissues, such as tumor tissue, including cell-penetrating peptides (CPPs), antibodies, and phage peptides (Fig. 2). Finally, protein nanoparticles themselves, as drug carriers, are among the new drug delivery systems. Abraxane, which contains albumin nanoparticles containing the anti-cancer drug Paclitaxel, has been approved by the relevant international organizations in recent years, and the significant annual growth trend of this drug and its effectiveness in treating various cancers has attracted the attention of many researchers Has attracted drug delivery [48–51]. Mathematical modeling plays an important role in facilitating the design of drug delivery systems by identifying key factors and molecular mechanisms of release [52–54].

### Nano medicine and proteins in the field of diagnosis

**Fig. 3:**
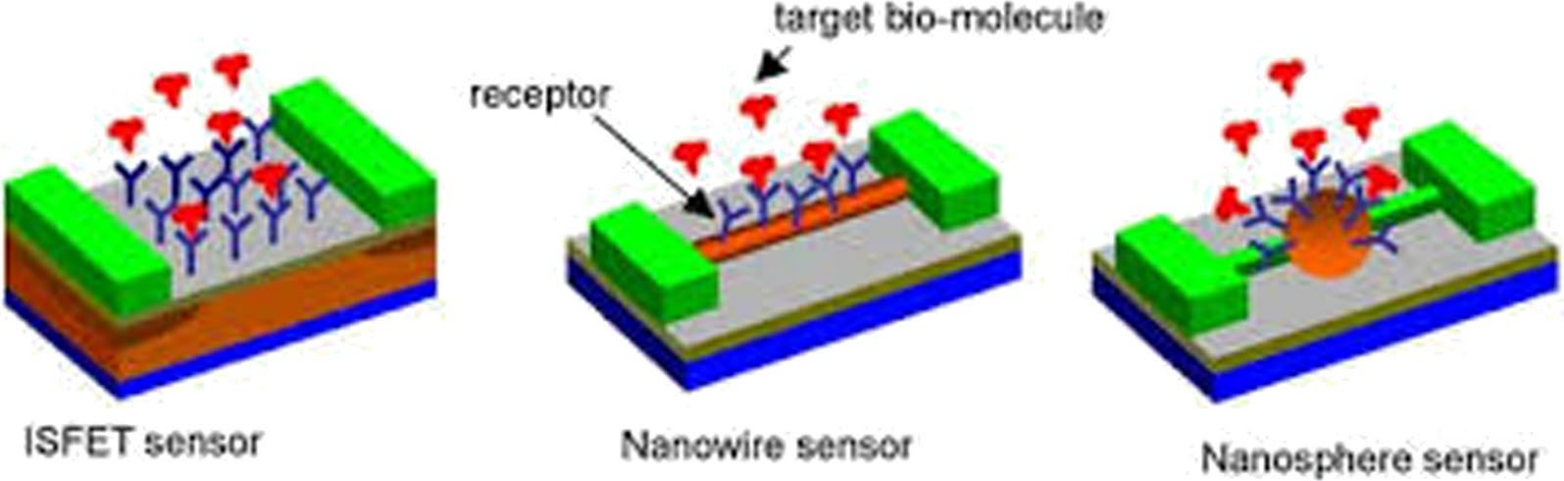
Nano sensors in the detection of proteins: different types of Nano biosensors are made to detect antibodies, antigens and protein biomarkers [55–57]

On the one hand, proteins are important factors in the diagnosis of diseases and on the other hand, they are used in the manufacture of sensors to diagnose other diseases [55–57]. Various Nano biosensors are being studied to detect a variety of proteins, antibodies, antigens and biomarkers, and factors such as antibodies and enzymes are being used to diagnose the disease [58–60]. For example, antibodies are used to detect a variety of viral diseases and the enzyme glucose oxidase is used in the manufacture of glucose Nano biosensors (Fig. 3).

## Advantages and disadvantaged of the general protein-based nanoparticle fabrication methods

**Fig. 4:**
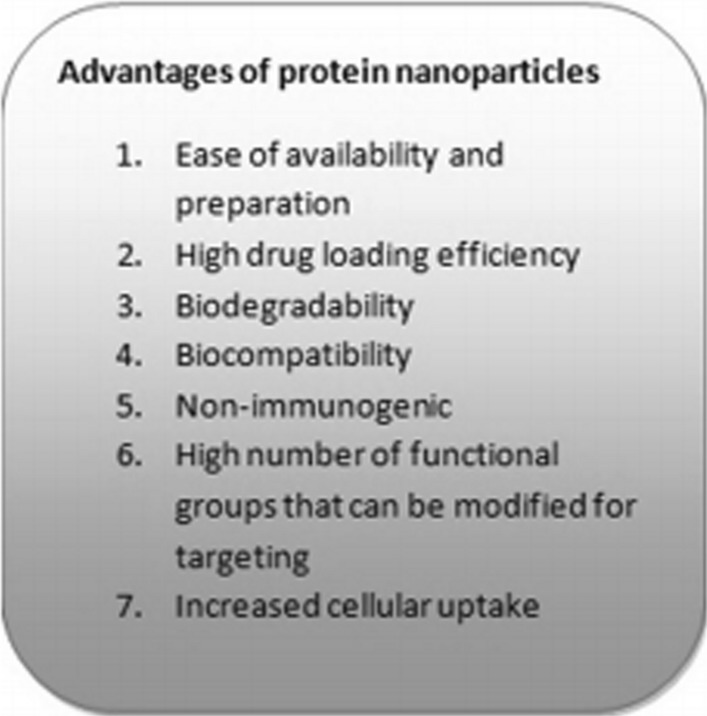
Advantages of protein nanoparticles in drug delivery [67]

The appropriate properties of protein nanoparticles have made them one of the important options in drug delivery and tissue engineering. The advantages and disadvantages of these fabrication methods are summarized in Table 2. Some of the most important of these benefits are [61–66]:

- Biocompatibility
  Proteins are among the major biomolecules that make up the body of all living organisms and therefore have little toxicity, especially compared to synthetic polymers [67–69]. By absorbing water and creating a space repulsion, proteins can increase the stability of nanoparticles [70–72] and also reduce the recognition of Nano carriers by the immune system (Fig. 4).
- Biodegradability
  These molecules are broken down in the body and the amino acids they produce are used by surrounding tissues to make proteins or produce energy [73, 74].
- Possibility of easy and cheap production
  Usually proteins are abundant in nature and are renewable sources by plants, animals, humans and other organisms. It is also possible to mass-produce a variety of proteins by recombinant protein production methods [75, 76].
- High drug binding capacity
  Proteins generally have many types of functional groups and therefore have the ability to bind and carry significant amounts of drug by different mechanisms such as electrostatic interactions, hydrophobic interactions, and covalent bonds [77–79].
- Proper uptake by cells
  Usually, proteins and polymer nanoparticles are removed by the cell by different mechanisms, for example, one of the effective factors mentioned in the anticancer drug Abraxane is the uptake of albumin nanoparticles by vascular endothelial cells [80–82].
- Targeting
  The structure and sequence of the protein and the presence of numerous different functional groups allow the binding of the drug to specific sites in the protein and the binding of different targeting ligands to the protein Nano carrier [83, 84].

**Table 2:** Advantages and disadvantaged of the general protein-based nanoparticle fabrication methods

Method	Advantages	Disadvantages	Refs.
pH Variation	Control for particle size Control secondary structure of protein Control for zeta potential Produces chemically and physically stable particles Experimentally simple	Post-fabrication drug loading is required Limited to small scale production	[35]
Spray-drying	Cost effective Experimentally simple Easily encapsulate hydrophilic drugs Useful for heat-sensitive samples Control for particle size	Limited to small scale production Challenging to incorporate hydrophobic drugs	[36]
Rapid Laminar Jet	Control for particle size Production of uniform particles Production of strong, stable particles	Possibility of coalescence Many parameters must be controlled for	[37]
Phase Separation	Specialized equipment is not required Particle size can be controlled by adjusting protein concentration Uniform particles are produced	Particle sizes are limited to 50–500 nm in diameter Organic solvents are required Limited to small scale production	[38]
Milling	Cost effective Large scale production is possible Control of nanoparticle size Experimentally simple	Heat is released during the process requiring chamber to be cooled Little control over nanoparticle shape Nanoparticles must be coarse	[39]
Polymer Chain Collapse	Properties of the nanoparticle can be easily controlled by selection of the precursor chain Production of particles with high stability Particles with improved spherical shape are produced	Particle size is limited to 5–20 nm in diameter Side reaction may be difficult to control	[40]

## Types of protein nanoparticles

### Animal proteins

#### Gelatin nanoparticles

**Fig. 5:**
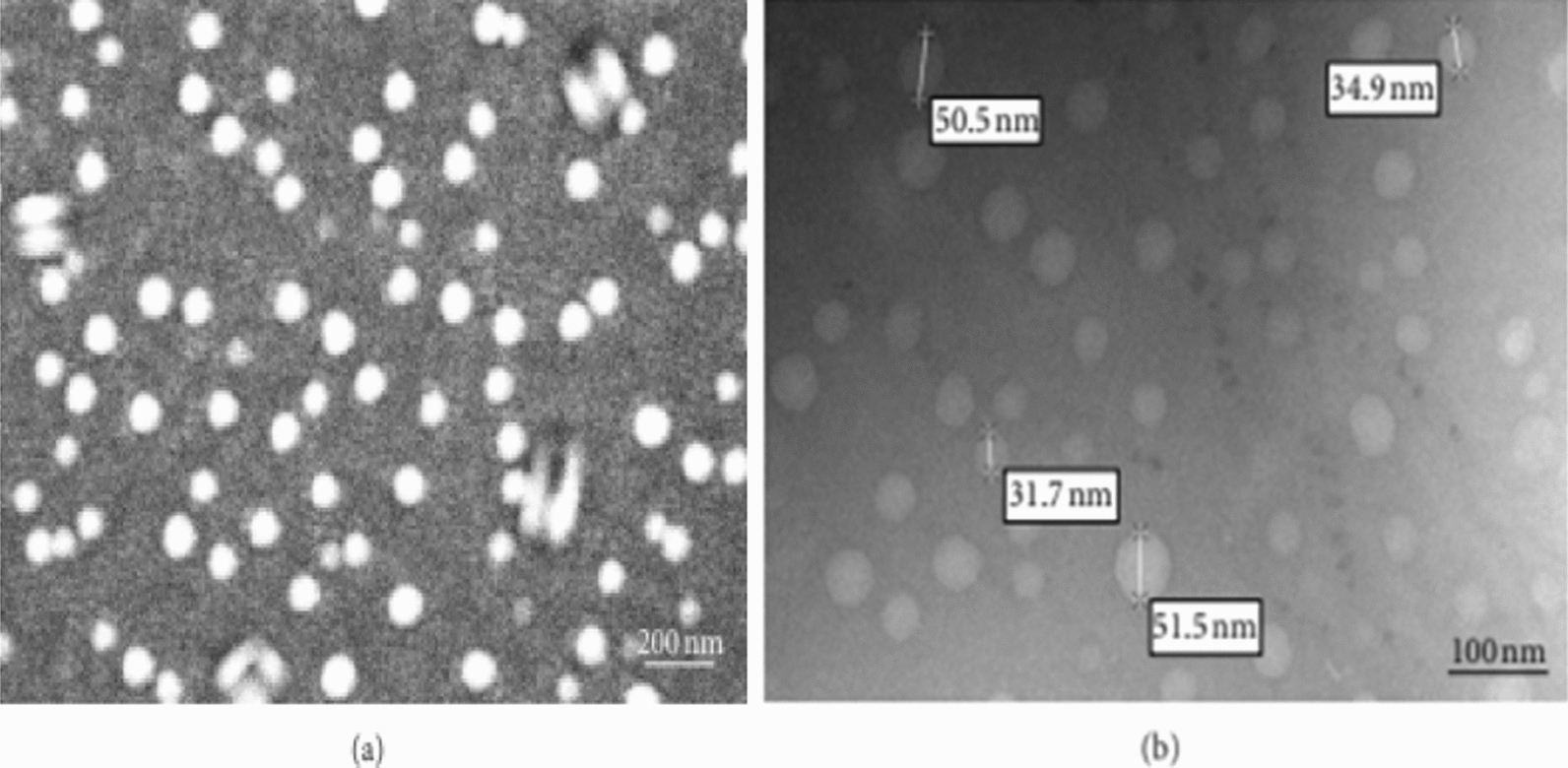
**a** SEM and **b** TEM images of gelatin nanoparticles [93–95]

Gelatin is a denatured protein derived from the acidic hydrolysis or collagen base of animals. This biomolecule has been used for many years in the pharmaceutical, cosmetics and food industries. Gelatin stimulates the immune system due to denaturation. Gelatin is a Polyampholyte compound and has cationic and anionic active groups and hydrophobic groups in a ratio of 1:1:1, so that the gelatin molecule has 13% positive charge (amino acids lysine and arginine), 12% negative charge (Glutamic and aspartic amino acids) and 11% of hydrophobic amino acids (leucine, isoleucine, methionine and valine) [85–87]. The rest of the structure is made up of glycine, proline and hydroxyproline. Gelatin is commercially available as both cationic gelatin and anionic gelatin. Cationic gelatin is obtained from type 1 pig skin collagen under acidic hydrolysis and anionic type from bovine collagen under hydrolysis [88, 89]. Gelatin is used in various drug formulations in systemic use. It is used clinically as a plasma volume enhancer as well as a stabilizer in protein formulations, vaccines and gelatin sponges such as gel foam. Gelatin contains the arginine-lysine-glycine sequence in its structure. The above sequence is an important sequence in many extracellular matrix proteins and plays an important role in cell binding and cellular messaging by binding to the beta subunit of integrin receptors at the cell surface [90–92]. This property is one of the important advantages of gelatin over polymers that lack cell recognition and binding sites. The active groups of gelatin make it possible to make a variety of chemical changes on it directly or by using different linkers, this feature is very important, especially when producing targeted drug delivery carriers and the possibility of attaching significant amounts of drug to carriers. Gelatin nanoparticles have been used to deliver various types of hydrophilic and hydrophobic drugs, including various anti-cancer drugs, anti-AIDS drugs, anti-malarial drugs, and analgesics, treatment of infectious diseases, muscle relaxants, anti-inflammatory drugs, and treatment drugs. Diabetes is a topical ophthalmic drug, inhibitor of protein synthesis, activator of tissue plasminogen, gene delivery and delivery of protein drugs and vaccines [93–95]. SEM and TEM images of gelatin nanoparticles is shown in Fig. 5, which clearly indicates the smooth And spherical nanoparticles with an average diameter of about 100–300 nm.

#### Synthesis of gelatin nanoparticles

**Fig. 6:**
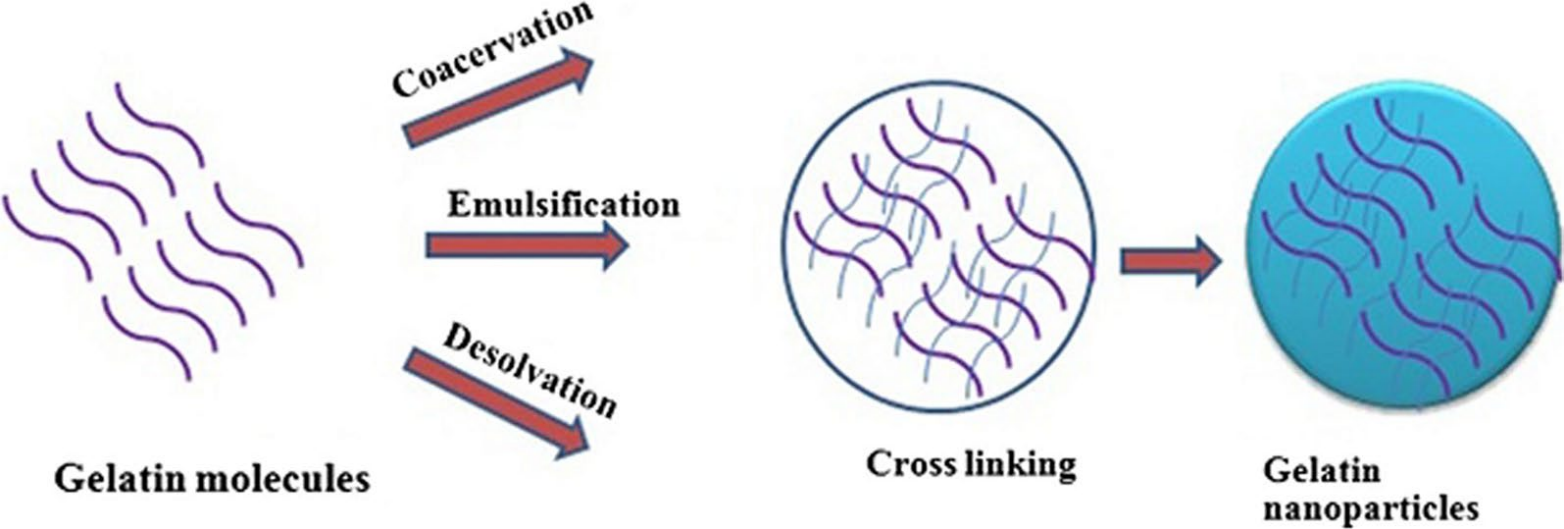
Synthesis of gelatin nanoparticles has been done in different ways, most of which in the final stages of synthesis, it is necessary to use a combination of linker (cross linking agent) to stabilize the nanoparticles

**Fig. 7:**
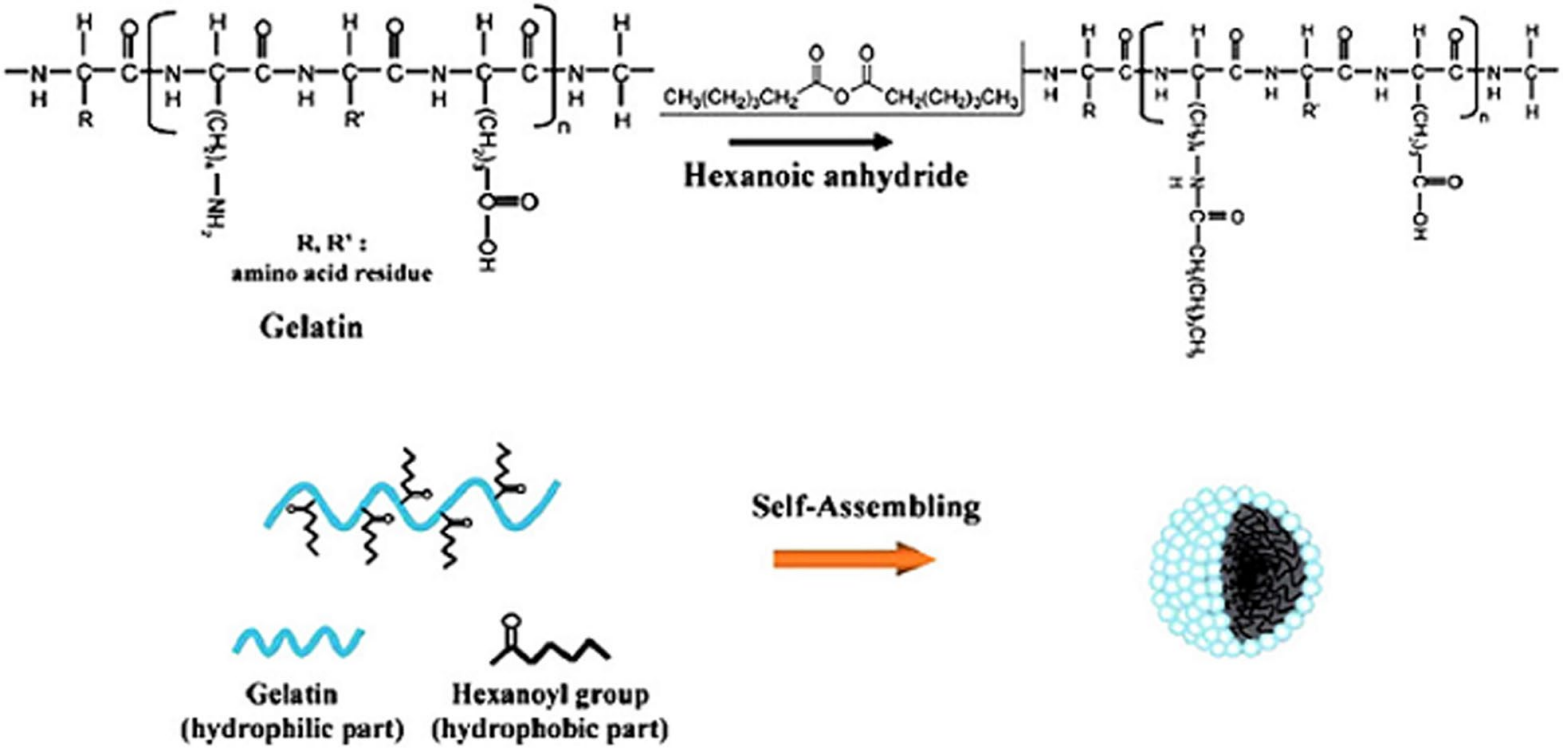
Synthesis of gelatin nanoparticles by self-assembly method: in this method, hexanoic anhydride molecules are first attached to it through the lysine roots in collagen, resulting in the formation of an amphipathic structure that spontaneously Nanoparticles accumulate [96]

Several methods are used to make gelatinous nanoparticles (Fig. 6). These methods include precipitation, phase separation, emulsion-solvent evaporation, self-assembly of gelatin molecules (which have been deformed by the bonding of chemical groups) or self-assembly of drugs and gelatin molecules, micro emulsion, and so on. The following is an example of the self-assembly of gelatin molecules (Fig. 7) [96].

#### Collagen nanoparticles

**Fig. 8:**
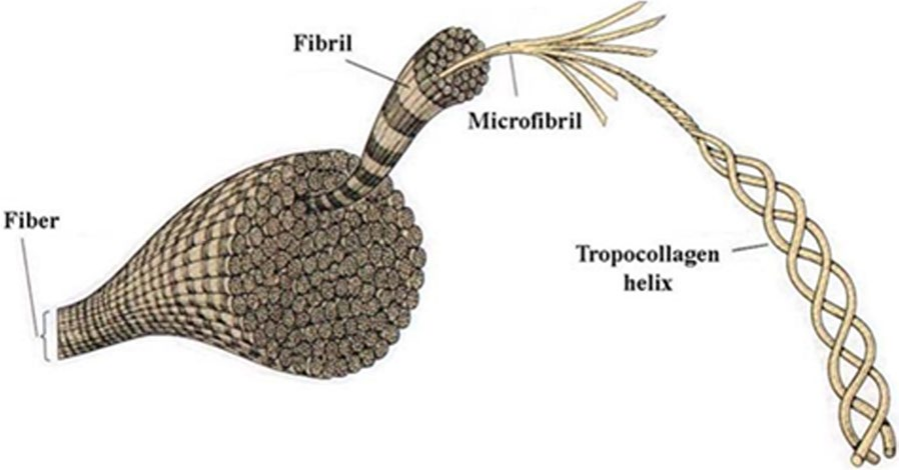
Structure of collagen fibers [101–104]

Collagen is a structural protein in the vertebrate body and is the most abundant protein in the mammalian body, accounting for 20–30% of the body's total proteins. The basic structure of collagen is tropocollagen three-stranded molecules that are twisted together in three spirals and connected by various nono covalent bonds [97–100]. Collagen is eventually produced by the formation of covalent crosslinks between tropocollagen molecules (Fig. 8). Due to its good biocompatibility and low stimulation of the immune system and biodegradability, this protein is widely used in medicine. Collagen nanoparticles are removed by the body's Reticuloendothelium system and can therefore increase the uptake of certain compounds, such as anti-AIDS drugs, into some cells, especially macrophages [101–104]. Collagen nanoparticles due to their small size with high contact surface, high absorption capacity and ability to disperse in water to form a stable and clear colloidal solution have been used as drug carriers for long-term release of antimicrobial and steroid drugs, especially in dermatology [105–107].

#### Milk proteins

Milk proteins are natural carriers of biologically active substances. Based on their structure, they can be classified into two categories [108, 109]:

1. The group of proteins with linear and flexible structure including caseins and proteins with spherical structure including whey proteins (whey) [110].
2. Beta-lactoglobin and alpha-lactoglobin are the main proteins in whey that have been studied to make drug Nano carriers. Among the characteristics of these proteins are their high resistance to breakdown by enzymes that break down proteins in the stomach [111, 112].

#### Casein

Casein is the main protein in milk. Its advantages as drug-carrying nanoparticles include low cost, easy access to its sources, high stability and nono Taxol [113, 114]. Many of the structural and physicochemical properties of caseins make it possible to use them as drug delivery systems. Some of these properties include the ability to bind to a variety of ions and molecules, exceptional stability and surface activity properties, excellent self-assembly and emulsification properties, and water-binding and gel-forming capacity. Caseins are not temperature sensitive, while whey globular proteins undergo denaturation and fundamental structural changes at temperatures above 70 °C [115–117]. The high tensile strength of casein films has made these proteins attractive in use as tablet coatings. Another important property of caseins is their protective effect, which is essential for the protection of sensitive cargoes. For example, casein with the ability to absorb strong light, especially in the wavelength range of 200–300 nm can protect its cargo against radiation, especially in the range of ultraviolet light [118–120]. The mentioned features suggest casein as a suitable candidate for building conventional and newer drug delivery systems such as nano-camels. However, limitations of caseins include immunosuppression and allergy concerns. It should be noted that casein in milk is absorbed as amino acids after decomposition in the gastrointestinal tract, but in cases such as direct intravenous injection of these proteins, the immune response to them should be considered [121–123].

#### Casein structure

**Fig. 9:**
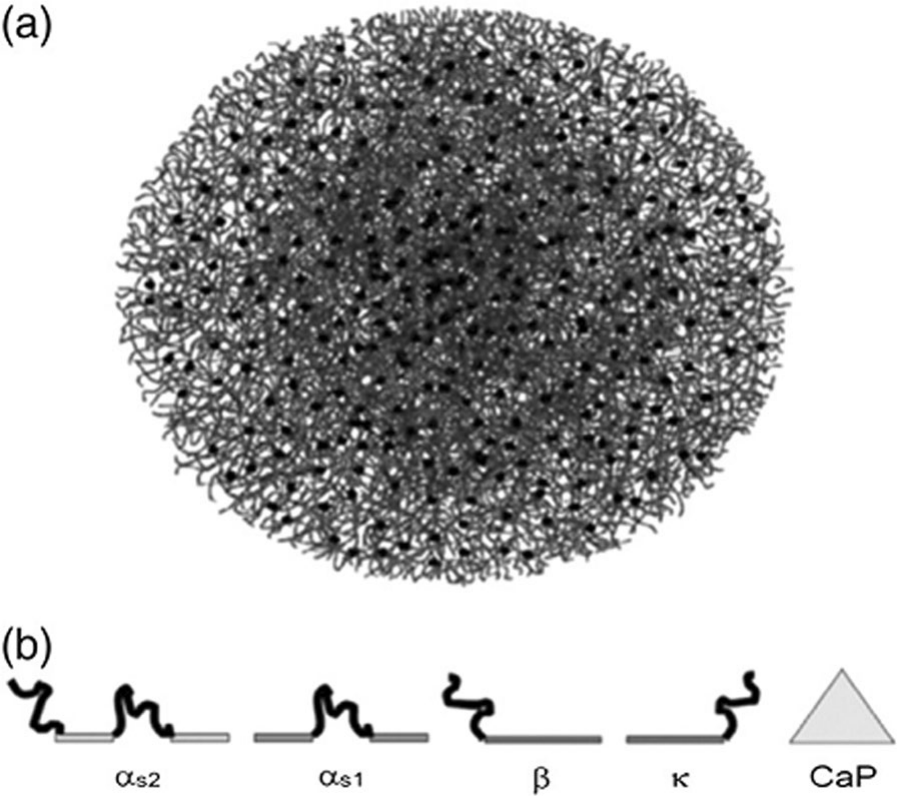
**a** Structure of casein micelles: filamentous casein monomers and black circles represent calcium phosphate Nano clusters. **b** Casein structural proteins: These proteins include hydrophobic regions (light linear parts) that react with each other and hydrophilic regions (dark ring parts) also interact with calcium phosphate Nano clusters [128, 129].

**Fig. 10:**
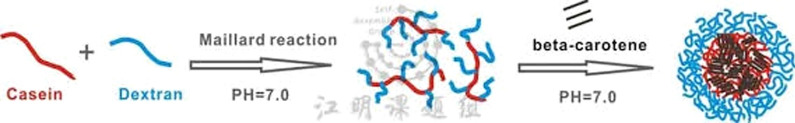
Cases of the casein and dextran copolymers have the ability to load and protect beta-carotene molecules [128, 129]

**Fig. 11:**
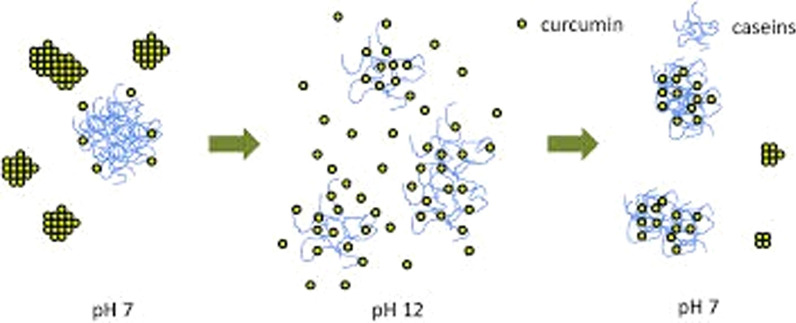
Production of casein nanoparticles containing curcumin: at pH 7 = curcumin molecules are complex (large particles) but with increasing to about 12 molecules of drug particles are dispersed and casein molecules are opened. By reducing the pH again to the range of 7, casein nano drates containing the drug curcumin are formed [128, 129]

Milk casein contains about 94% protein and 6% low molecular weight compounds called colloidal calcium phosphate. These Phosphoprotein have a molecular weight of 19–25 kDa and an isoelectric pH of 4.6–4.8. Caseins contain multiple roots of hydrophilic and hydrophobic amino acids, and are therefore dual-protein proteins capable of producing block copolymers with a high tendency to self-regulate micelles in the range of 50–500 nm (average 250 nm) [124]. These spherical micelles have a hydrophobic inner part whose outer surface is surrounded by a layer of hydrophilic casein kappa (κ) that stabilizes the micelles by creating an electrostatic and spatial repulsion between the micelles (Fig. 9). In fact, casein micelles in milk are natural nano carriers that are responsible for transporting and supplying amino acids and calcium phosphate from mother to baby [125–127]. These micelles are very stable and maintain their structural stability while performing various processes on milk to prepare a variety of dairy products. More recently, casein or copolymer micelles have been used with other polymers to transport hydrophobic cargoes, effectively inhibiting vitamin D and omega-3 unsaturated fatty acids and beta-carotene (a precursor to vitamin A) against degradation and oxidation by Protect from ultraviolet light (Fig. 10). Casein nano micels have been used as carriers of various anticancer drugs such as curcumin, mitoxantrone, vinbelastin, docetaxel, and paclitaxel (Fig. 11). Beta casein is a candidate for targeting gastric tumors due to its degradability in the stomach. By degrading beta-casein nanoparticles in the stomach by the enzyme pepsin, paclitaxel is released from them and effectively reduces the growth of gastric cancer cells. Nano mysel protects the drug by protecting the drug inside it and preventing the drug from being released before it reaches the stomach, as well as preventing its toxic effect on higher areas of the gastrointestinal tract such as the mouth and esophagus. Gels from this protein are sensitive to pH changes and can be used in the construction of intelligent drug delivery systems. Another potential of casein nanoparticles is their ability to be lyophilized without the need for cryo-protectants when preparing drug formulations [128, 129].

#### Silk fibroin

Silk is a natural protein polymer produced by the larvae of some insects, such as silkworms and spiders. Due to their good biocompatibility, these proteins are being studied in drug delivery and tissue engineering. The main components of silk are the fibrin linear protein in the nucleus and the adhesive protein such as serein, which encapsulates the fibrin nucleus [130]. Among the potentials of these proteins in making nanoparticles and scaffolds with low decomposition rate is their self-assembly ability and mechanical properties. Fibroin has a lower inflammatory response at the site of degradation than widely used biocompatible synthetic polymers such as polylactic acid. Fibroin nanoparticles have the ability to protect proteins and peptides such as conjugated insulin and vascular endothelial growth factor in blood serum and solution containing the enzyme trypsin (enzyme that breaks down proteins in the human stomach) and increase the duration of active release of these compounds [131–133].

#### Elastin

**Fig. 12:**
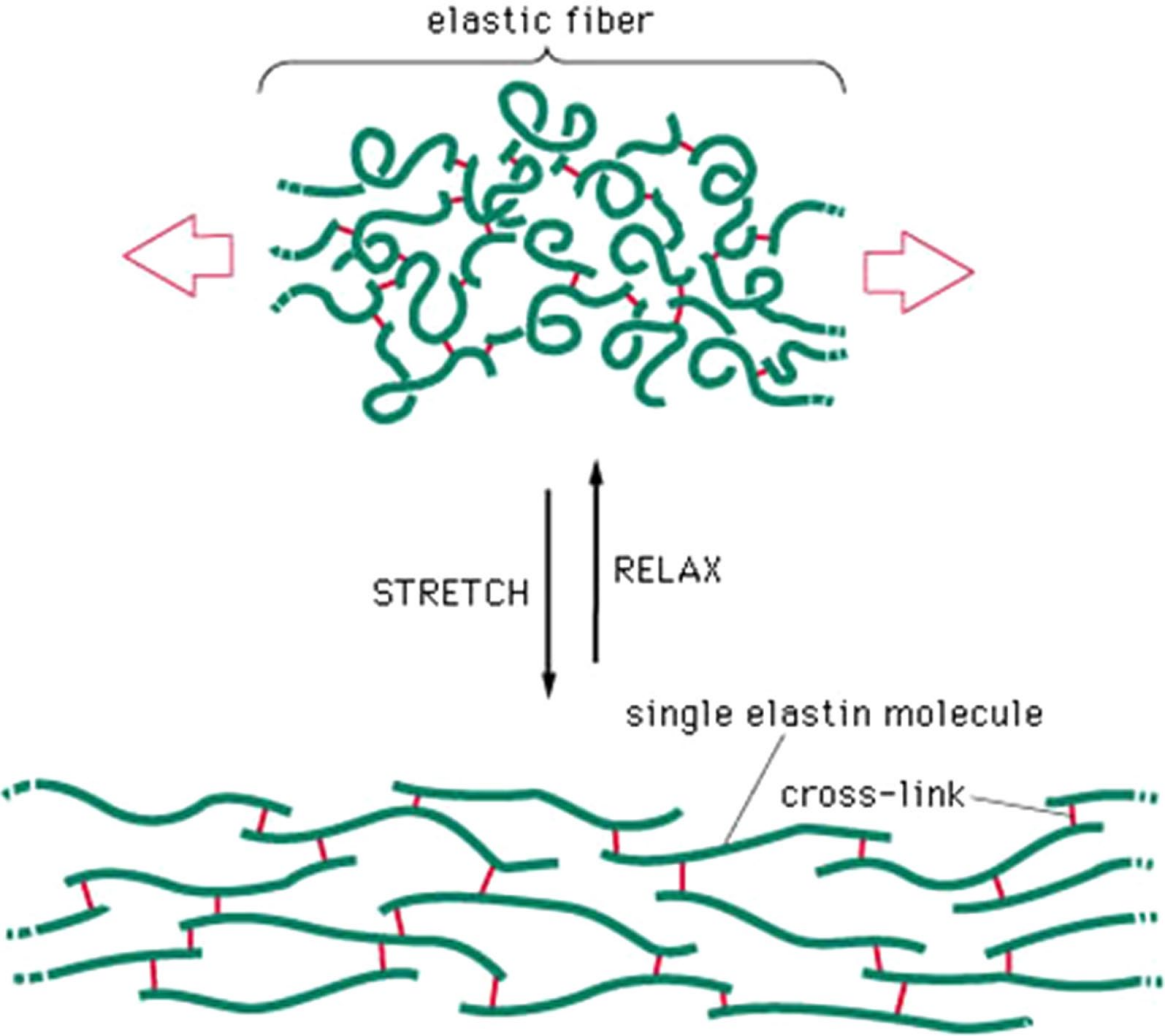
Elastic fiber structure. Changing the pattern of bonds between elastin molecules causes elastic properties and flexibility in elastin fiber and tissue [139–142]

**Fig. 13:**
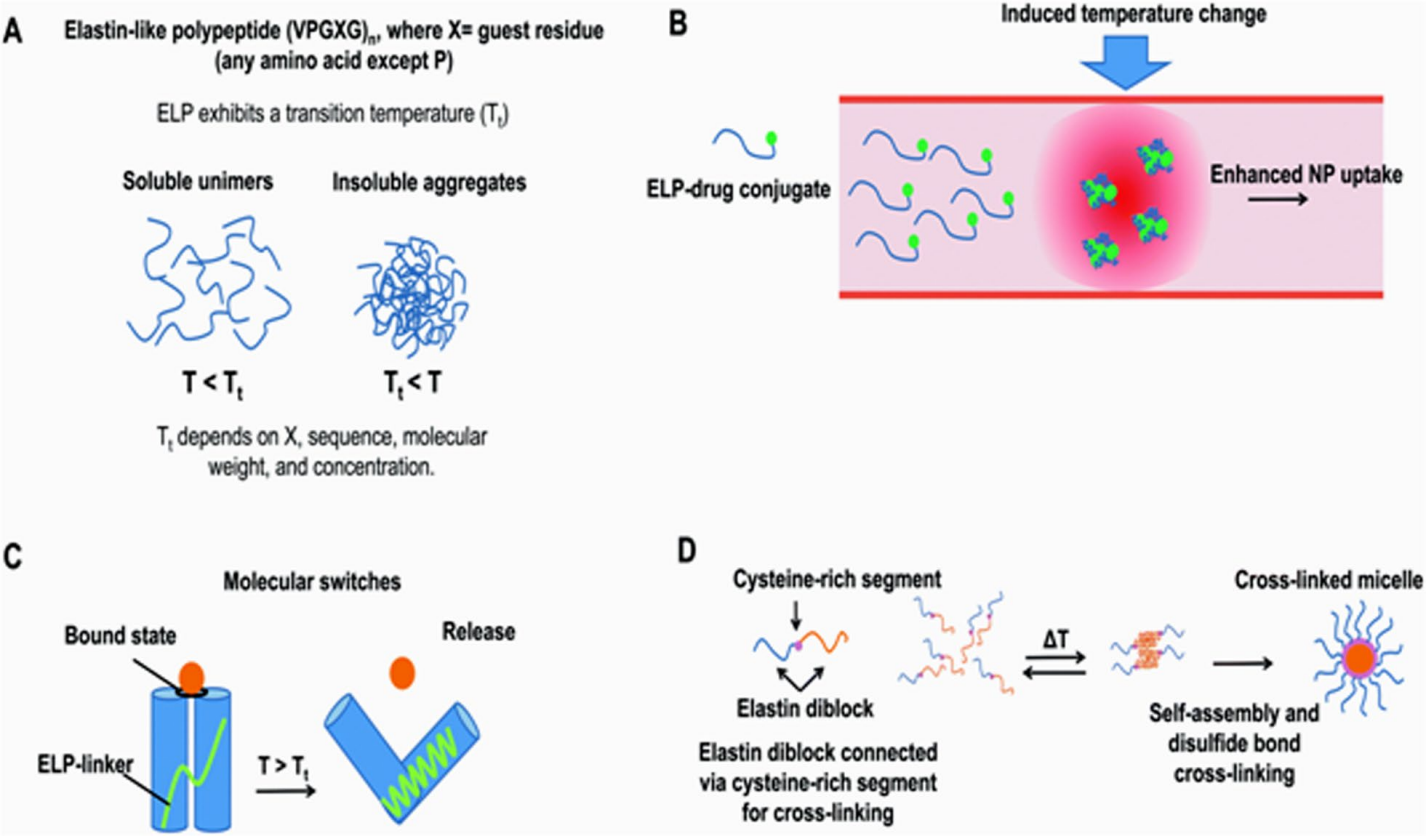
Temperature responsive ELP nanoparticles. ELP. **A** with n-repeating peptide sequence (Val-Pro-Gly-X-Gly) is a temperature-sensitive polypeptide that is insoluble in water above the transfer temperature (Tt) and soluble in water at the bottom. **B** Drug-ELP conjugates in heated tumor tissue can aggregate to produce drug-containing ELP nanoparticles that will increase in size depending on their uptake into the target tissue (EPR mechanism). **C** Molecular thermal switches based on ELP nanoparticles. **D** Production of temperature-sensitive micelles by ELP molecules containing cysteine-rich sequences [139–142]

Elastin is the predominant protein in the extracellular matrix of arterial walls. This protein plays an important role in creating elastic properties and flexibility in the arteries when blood pressure changes, as well as in many other tissues of the body such as the lungs, skin and ligaments [134, 135]. In their natural environment, the components of elastin first converge in the form of a water-soluble precursor molecule called their tropoelastin, and then these precursors combine to form elastin fibers by forming covalent cross-links (Fig. 12). Genetic engineering techniques and the production of recombinant proteins have made it possible to make elastin-like polymers (ELPs) [136]. The basic structure of these proteins is similar to the repetitive sequences found in elastin, but genetic engineering methods have made it possible to add specific sequences and create the desired properties of researchers in these polymers [137]. Due to their similarity to natural elastin in the body, the immune system does not react to them and they have the ability to escape from the immune system [138]. The possibility of designing and manufacturing elastin-like proteins by genetic engineering techniques gives several benefits to these proteins and their nanoparticles, including the ability to achieve appropriate pharmacokinetic properties, the ability to precisely control molecular weight, and the production of single-size polymers. (monodisperese), the ability to attach multiple drug molecules to them and the ability to attach nanoparticle targeting agents to specific tissues or locations in the body [139–142]. Also, a variety of polymers designed with these methods have the ability to change the phase rapidly in response to temperature changes (Fig. 13).

## Plant proteins

The use of plant protein nano carriers is a new approach in drug delivery. Unlike animal protein nano carriers, plant proteins such as zein and gliadin have a longer drug release ability due to their hydrophobic nature [143–145]. Also, due to high hydrophobicity, stable nanoparticles of plant proteins may be produced without the need for chemical and physical treatments and the use of chemical linker molecules, which are often used in the manufacture of animal protein Nano carriers [146–148]. Vegetable proteins are widely available and are much cheaper than animal proteins. They are also not at risk of transmitting animal diseases to humans, such as bovine insanity. The presence of different functional groups in these proteins makes it possible to change the surface of the resulting nanoparticles to regulate the physical and chemical properties and bind the targeting agent [149–152].

### Zein

**Fig. 14:**
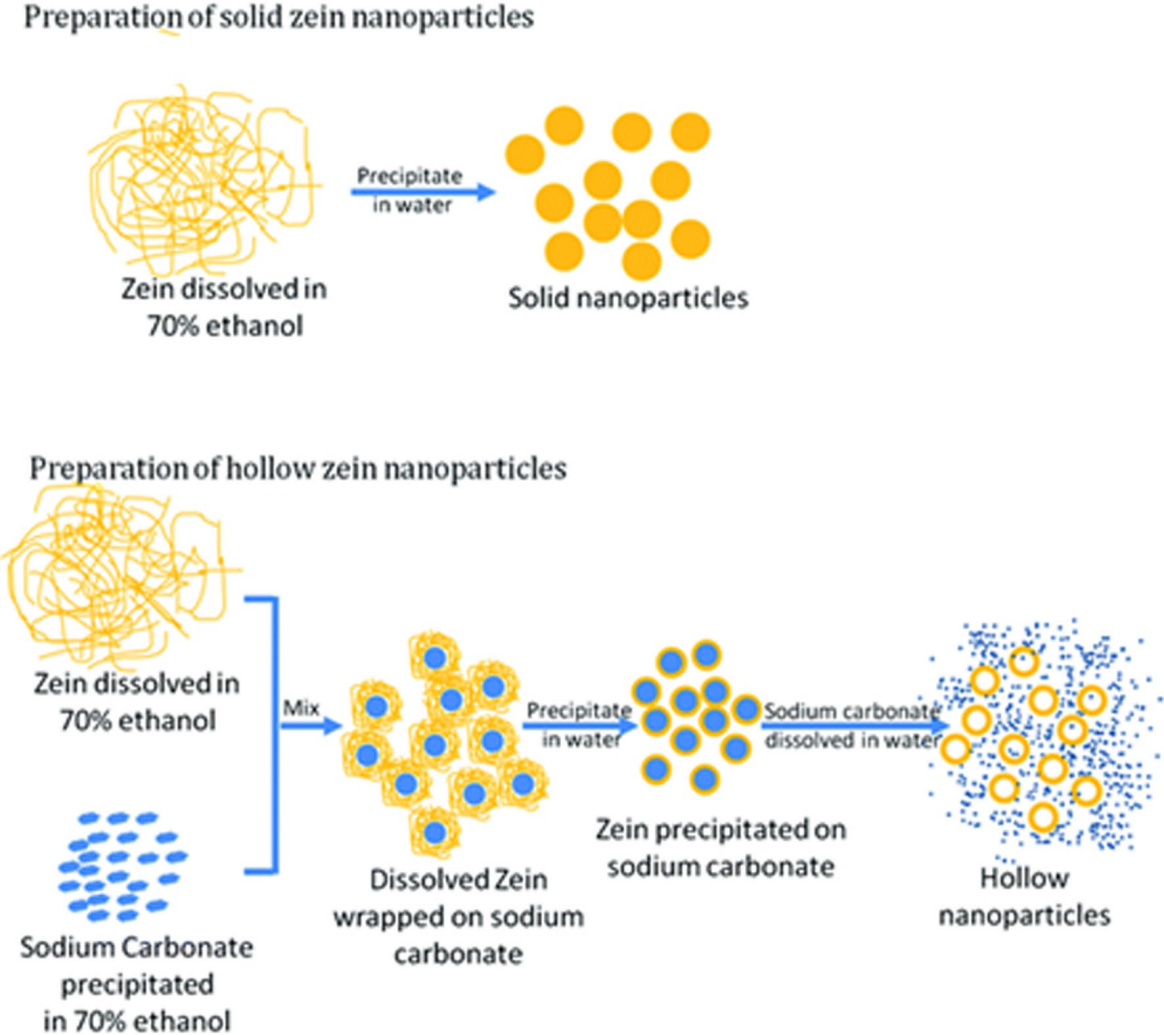
Production methods of cup nanoparticles (nano sphere) and hollow nanoparticles (nano capsules) of zein [155–157]

Saddle is a water-soluble, alcohol-soluble protein with a molecular weight of about 40 kDa, which is found mainly in cereals [153]. 75% of its amino acids are hydrophobic and 25% are hydrophilic. Because zein is a natural protein and has good biodegradability, it has been approved by international organizations for use in the food and pharmaceutical industries [154]. It is also used as a degradable coating in foods and drugs today due to its low water absorption, high temperature resistance and suitable mechanical properties. This protein forms nanoparticles in the alcoholic-aqueous solution in the range of 150–550 nm (Fig. 14) and due to high hydrophobicity is considered as a controlled drug delivery system for hydrophobic drugs. Also, the special shape of bricks like this protein enables the ability to carry hydrophilic compounds such as heparin, 5-fluorouracil, and doxorubicin and control their release and improve their effect [155–157].

### Gliadin

Wheat gluten is a complex containing proteins and carbohydrates, of which proteins are the main components. These proteins include glutenin and gliadin. Isolation and detection of these proteins is done with 70% alcohol. Glutenin is an alcohol insoluble protein with a molecular weight of 106 kDa. Gliadin is a set of proteins that are separated from alcohol by 70% of gluten and have a molecular weight in the range of 25–100 kDa. The structure of these proteins also contains large amounts of the amino acid glutamine (about 40%) [158, 159]. Gliadin has little solubility in aqueous solutions [160–162]. Because they are similar to creatine, these proteins are rich in proline and have the ability to interact with the skin's creatine epidermis and have the potential to produce skin formulations. Various studies have shown the ability of Gliadin nanoparticles as drug release control systems for hydrophobic compounds and amphiphilic compounds such as vitamin A, vitamin E, amoxicillin [163, 164]. One of the useful properties of Gliadin is its high ability to bind to the body mucosa [165, 166]. Due to the presence of glutamine and hydrophobic amino acids in its structure, Gliadin on the one hand gives abundant hydrogen bonds with the mucous layer of the mucosa and on the other hand can interact with the cell membrane by hydrophobic interactions. For this reason, Gliadin nanoparticles have shown good potential in the preparation of oral formulations, especially for the treatment of gastric diseases such as gastric ulcers. Due to their interaction with gastric mucus, these nanoparticles increase bioavailability and increase the release time of the drug, resulting in the effective removal of Helicobacter pylori (the cause of gastric ulcer) from the mucosa of this organ [167, 168].

### Lectin

**Fig. 15:**
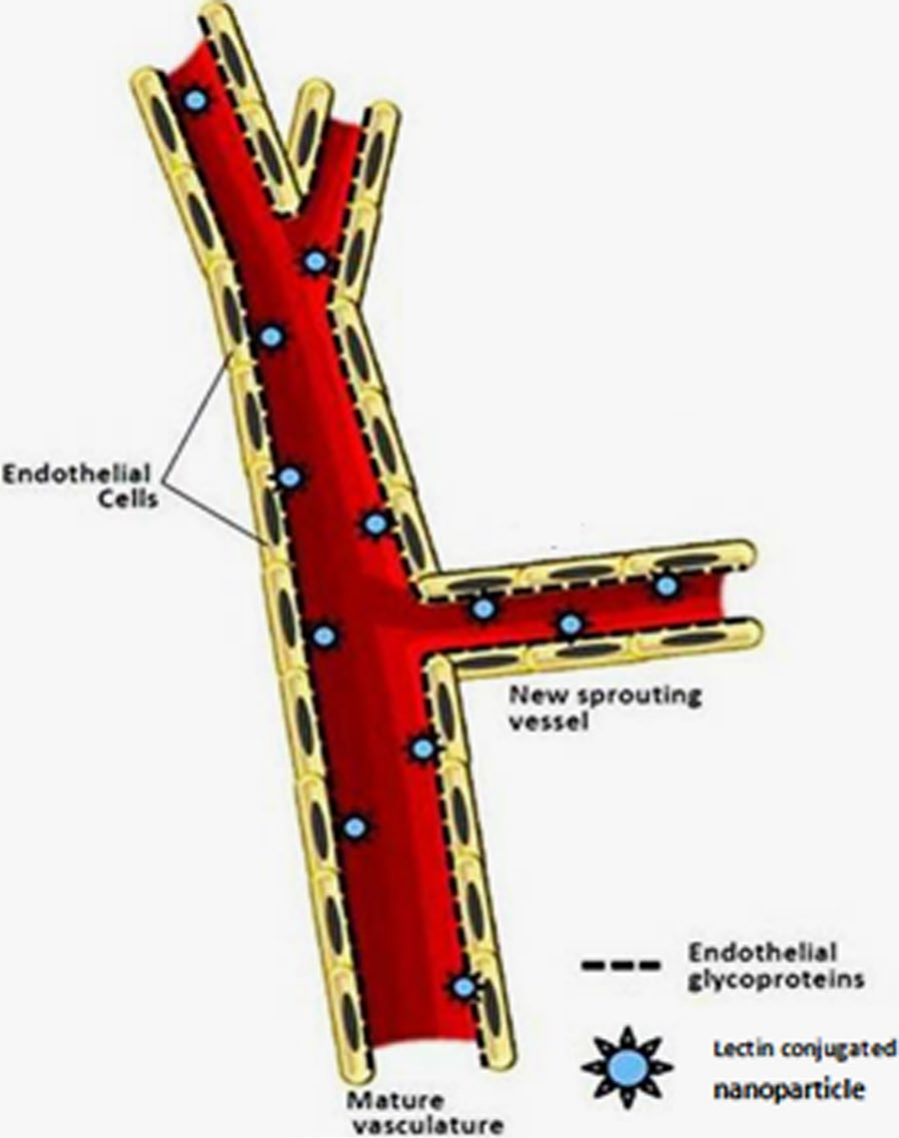
Nanoparticles coated with a variety of lectins have the ability to bind to glycoproteins of the luminal surface of vascular endothelial cell membranes and can be used in targeted drug delivery [186–189]

**Fig. 16:**
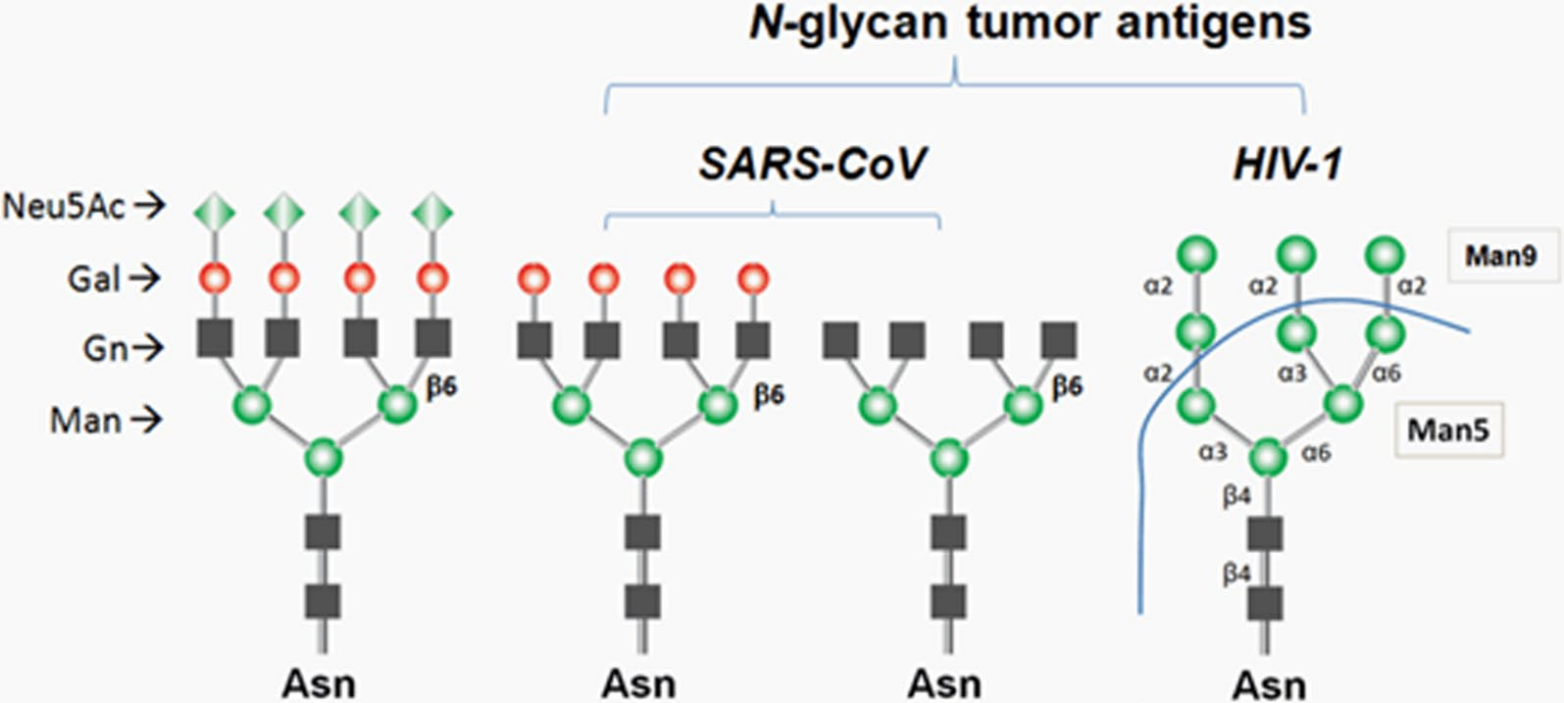
Some sugar sequences in tumors and viruses are different from those in normal cells. These sequences can be used as target lectin antigens in targeted drug delivery [186–189]

Lectins are a diverse group of glycoproteins or proteins capable of binding to carbohydrates. Wheat germ agglutinin (WGA) is one of the most popular plant lectins that is of great interest. This protein has high stability, low toxicity and immunogenicity, resistance to proteolytic degradation as well as specific identification and binding site to glycosylated components of intestinal mucosa and therefore can improve the absorption of oral drug formulations [169–172]. In the last two decades, lectins in two the main area has been considered by the pharmaceutical industry. The first is to improve the absorption of existing drugs with low bioavailability and the second is to prepare targeted drug formulations in the treatment of cancers [173–176]. In addition, several types of lectins, including WGA, have shown significant antitumor effects by inducing apoptosis in cancer cells. Numerous proteins and phospholipids are present in cell membranes attached to different oligosaccharide roots and have the ability to bind specifically [177–179]. Lectins to these sugar roots at the cell surface are the basis for targeted drug delivery by lectins (Fig. 15). Because different cells produce and display different types of oligosaccharide chains on their surface, cancer cells also often show different oligosaccharide patterns than normal cells of the same type, so different lectins can be used as Carriers are used to deliver the drug to different tissues and cells (Fig. 16). Accordingly, many studies have been conducted in this field to cover different types of nanoparticles with lectins and to produce targeted drug delivery systems. Lectins can be introduced as the second generation of bioadhesive enhancers, because in addition to the release of nanoparticles from the mucosal layer of the mucosa by various mechanisms such as clathrin-dependent endocytosis and endocytosis by the Caveola pathway causes increased cell uptake of drug formulations [180]. Lectins with the ability to bind to carbohydrates on the surface of Helicobacter pylori can also increase the effectiveness of treatment [175, 176, 181]. Lectins are also useful in the development of oral vaccines. Nanoparticles containing pathogenic antigens and coated with lectins targeted to the surface of Peyer’s patches in the intestine enhance the immune response of the oral vaccine [182, 183]. Aged plaque cells are cells that display antigens and components of the immune system located in the gut [184, 185]. The ability to bind lectins to the mucosa is used not only for the gastrointestinal mucosa but also to improve drug delivery through non-oral pathways such as the nasal mucosa, vagina, lungs, eyes, as well as crossing the blood–brain barrier. odorranalectin, the smallest member of the lectin family, is less immunogenic than other lectins and has the ability to specifically detect and bind to L-fucose. This sugar is abundant on the surface of cells lining the nasal mucosa. Nanoparticles containing this lectin have increased nose-to-brain delivery to the brain [186–189].

### Soy proteins

Soybeans are currently one of the most abundant sources of plant protein. The fortified form of soy protein is called soy protein isolate (SPI). Soy protein extract is a balanced combination of polar, non-polar and pregnant amino acids that allows its use in a variety of drugs. The main components of SPI are glycinin with a molecular weight of 360 kDa and beta-kan glycinin with a molecular weight of 180 kDa. In aqueous solution, SPI proteins form a spherical structure consisting of a hydrophilic shell and a hydrophobic nucleus. Addition of precipitating agent (dissolvent = solvent) or linker molecules accumulate in different structures such as microsphere and hydrogel. By changing the amount of linker agents are added and as a result the percentage of binding in the resulting particles can be particle decomposition pattern to achieve the appropriate pattern Drug release altered Soy protein nanoparticles can be obtained from fresh SPI by desolvation or coacervation [190–193].

## Protein cages

### Viral protein cages

**Fig. 17:**
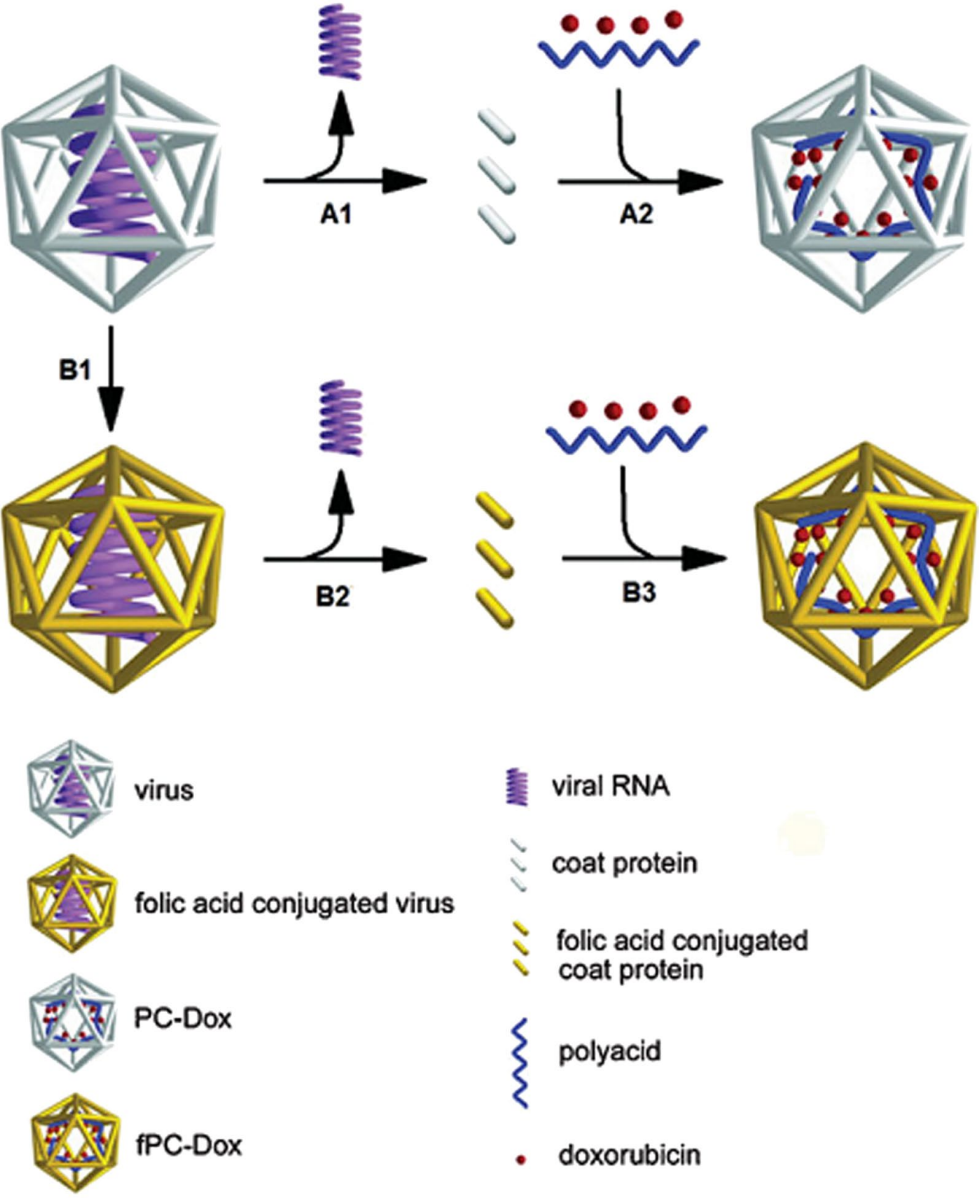
Mechanism of loading doxorubicin chemotherapy into the protein cage alone (A1, A2) or simultaneously with the binding of the targeting agent (folic acid) to the protein cage (B1, B2, B3) [195–198]

**Fig. 18:**
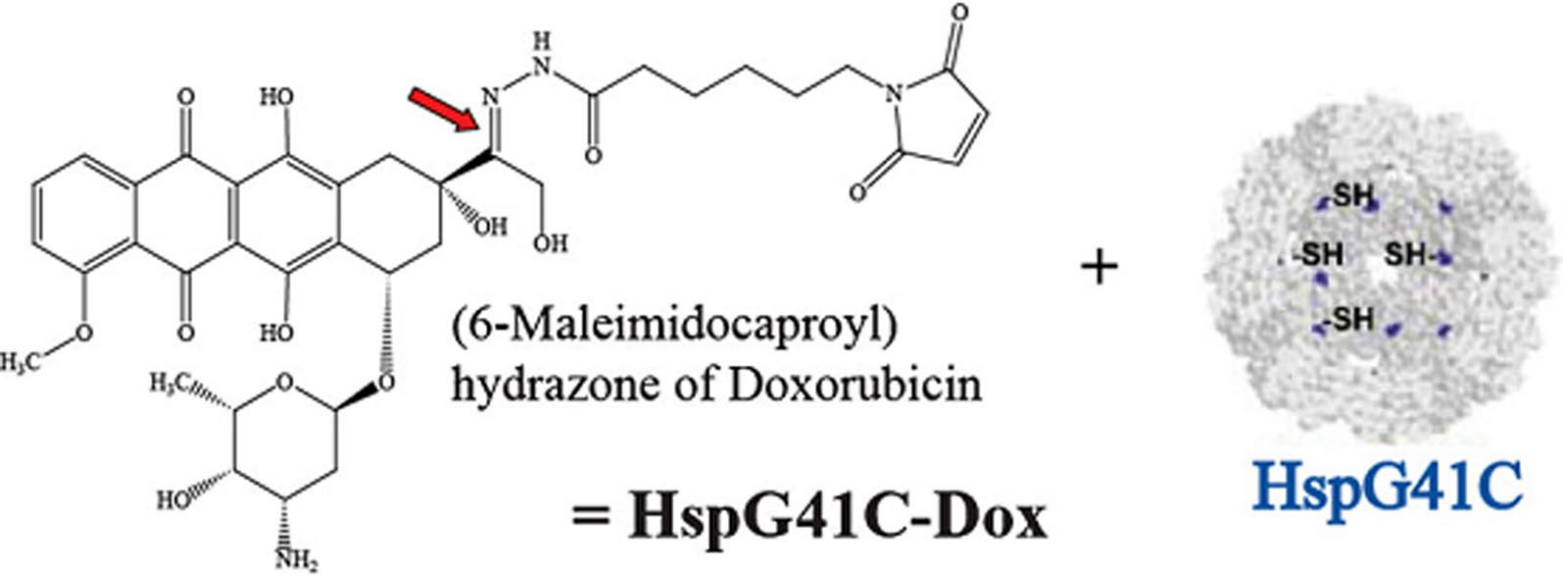
By genetic engineering methods, thiol (SH) cysteine roots are added to recombinant heat shock protein (HspG41C) nanocages. The resulting nano cages are able to bind to a significant number of doxorubicin drugs [195–198]

Protein cages are structures derived from viruses or virus-like particles. These particles often range in size from a few nanometers to a few tens of nanometers. Virus cages are actually the structural shell or capsid of viruses without their nucleic acid content. The shape, size and stability of virus cages depend on the type of virus. These cages consist of a limited number of subunits that accumulate in the form of porous nano spheres. In this structure, three distinct areas are significant, which are the inner and outer surface of the cage and the distance between the subunits. All three regions can be modified by chemical methods or genetic engineering methods (by changing the nucleotide sequence of subunits) without changing the structure of the cage, for use in applications in medical diagnosis and treatment [194]. Protein cages usually have good stability in different chemical environments (to cause chemical changes on them). In this way, a protein cage can be designed that has the ability to perform several operations simultaneously, such as drug loading, imaging agent, and cage targeting agent to a specific cell or tissue (Fig. 17). For example, by adding the amino acids cysteine and lysine to the cage by genetic engineering methods, it is possible to attach different drugs, imaging agents and fluorophores to the cage (Fig. 18). Another distinctive feature of protein cages compared to other protein structures is the uniform size of the cage. This property makes it possible to load relatively specific amounts of drug into these nanoparticles, which is an important pharmacokinetic feature of a drug formulation. The resulting protein cage is naturally stable in many physiological environments and protects drugs and therapeutic agents against chemical and enzymatic degradation. Cancer chemotherapy is another potential application of protein cages [195, 196]. Due to their size (tens of nanometers), nanometer cages do not pass through the endothelial layer of normal vessels, so they have a longer half-life in the bloodstream, but these cages are smaller than the pores of tumor tissue vessels (Fenestrate) and can enter and Adhesion to the surface of cells and tumor tissue can effectively inject significant amounts of chemotherapy drug into tumor tissue. The small size of the cages also helps these particles escape from the macrophage cells of the liver tissue [197, 198].

### Non-viral protein cages

**Fig. 19:**
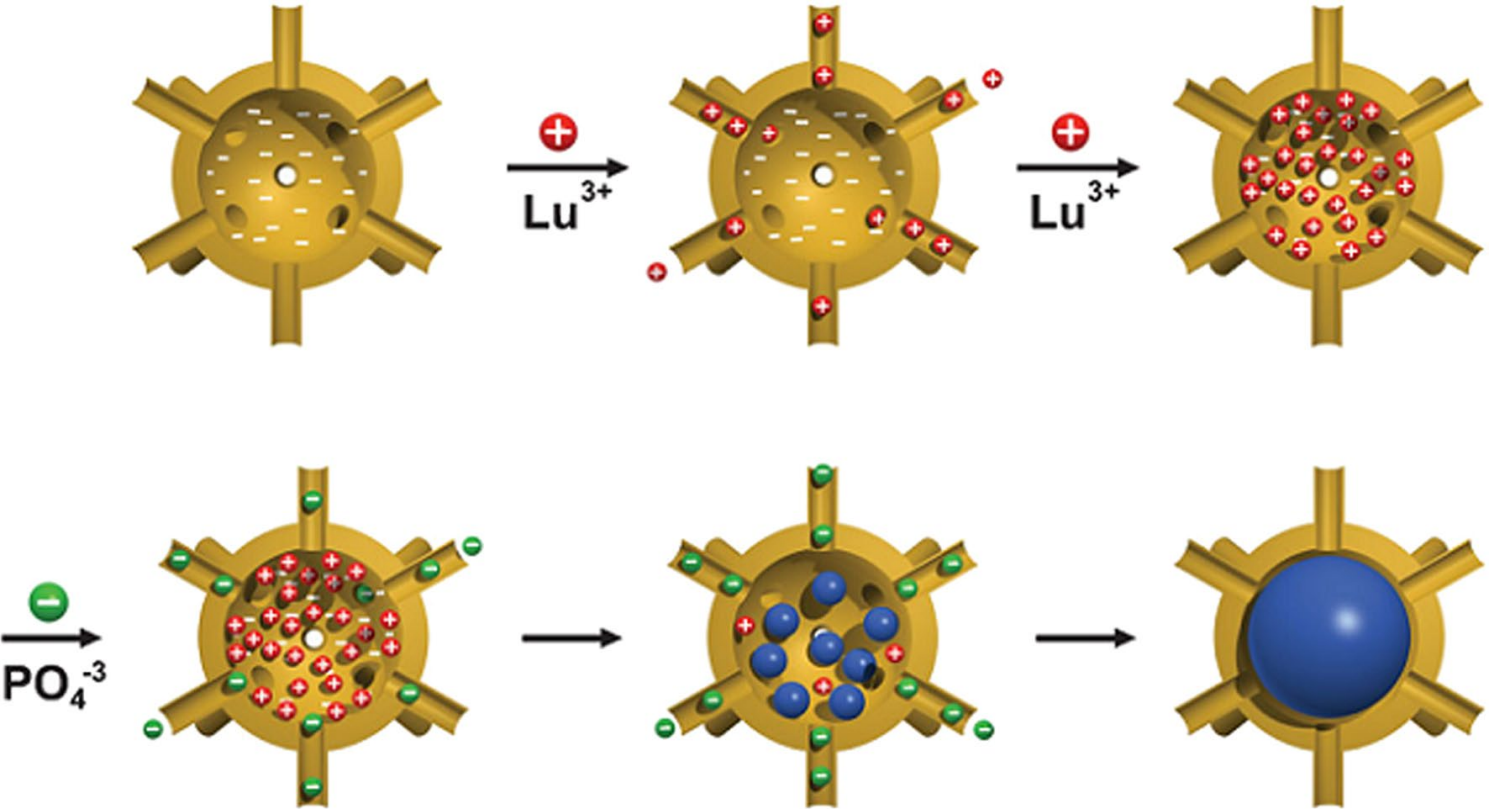
Loading of luteum phosphate into ferritin nano cages by diffusion mechanism [199]

In addition to viruses, ferritin/apophytin protein cages and small heat shock shock protein are among the protein cages. Ferritin cages consist of 24 subunits that are arranged in the form of hollow structures with a diameter of 12 nm and an internal space of 8 nm. Normally in the body, this space is used to store about 4500 iron ions. In this structure, there are 14 channels for the exchange of materials between the cage and the external environment. These nano cages have been used to carry various molecules and ions [199]. Lutetium-177 (Lutetium-177) is a radioactive material that is highly regarded for imaging and radiotherapy applications in nuclear medicine due to its good half-life (6–7 days) and beta and gamma radiation. Numerous cancers, such as neuroendocrine, pancreatic, prostate, lung, bone marrow, and leukemia tumors, have been shown to be able to load large amounts of this radioactive substance into the body and increase its stability in the body. The higher dose of radiation to the tumor tissue will be during radiotherapy (Fig. 19).

## Disadvantages and limitations of protein nanoparticles

Despite the various advantages mentioned regarding the use of protein nanoparticles in drug delivery and tissue engineering, there are some limitations to these natural polymers in their use in the pharmaceutical and medical industries, some of the most important of which are [200–202]:

1. Proteins are natural polymers and most of them are heterogeneous mixtures of different sizes with different molecular weights. This feature reduces the possibility of reproducibility and the possibility of differences in product characteristics at different times (batch to batch variation) during mass production and industrial pharmaceutical uses. To overcome this problem, researchers are looking to produce recombinant proteins using genetic engineering techniques [203, 204]. The proteins produced by these methods are single in size and have a fixed and specific molecular weight. By designing their structure, it is possible to connect different groups to their surface, such as targeting factors, and also to adjust the rate of decomposition and release of the drug by them. In this regard, various types of proteins have been produced for use in drug delivery, including elastin proteins such as ELPs, recombinant human serum albumin (rHSA) and recombinant gelatin. It should be noted that the production of proteins by genetic engineering methods leads to an increase in the cost of products.
2. Immunogenicity: the human body has shown an immune response to foreign proteins and different degrees of immunogenicity are among the limitations of protein nanoparticles. However, when intravenous injection of albumin, gelatin, casein and zein nanoparticles [205–208], little immune response was observed [209].
3. Achieving a proper release pattern: since proteins are often hydrophilic molecules, most of them are not able to release the drug for a long time and their nanoparticles swell when they enter the body by absorbing water and the drug spreads rapidly outside. Therefore, chemical linker molecules such as formaldehyde and glutar aldehyde are usually used to stabilize their structure when preparing protein nanoparticles [210–214]. These interface molecules are often toxic, so one of the areas of active research in the field of protein nanoparticles is the achievement of suitable and non-toxic linkers. Also, plant proteins with hydrophobic nature have shown promising results in the production of protein nano carriers [215–219] with long-term release capacity.
4. The possibility of transmitting animal diseases such as bovine insanity to humans when using animal protein sources to produce nanoparticles [220–223].

## Advantages of albumin nanoparticles as a drug delivery system

Formulations based on albumin nanoparticles have several advantages, some of which are due to the use of albumin as a structural unit of nanoparticles and others related to the properties of these nanoparticles, and Mathematical modeling plays an important role in facilitating the design of drug delivery systems by identifying key factors and molecular mechanisms of release [224–227]. Which are mentioned below [228, 229].

**Fig. 20:**
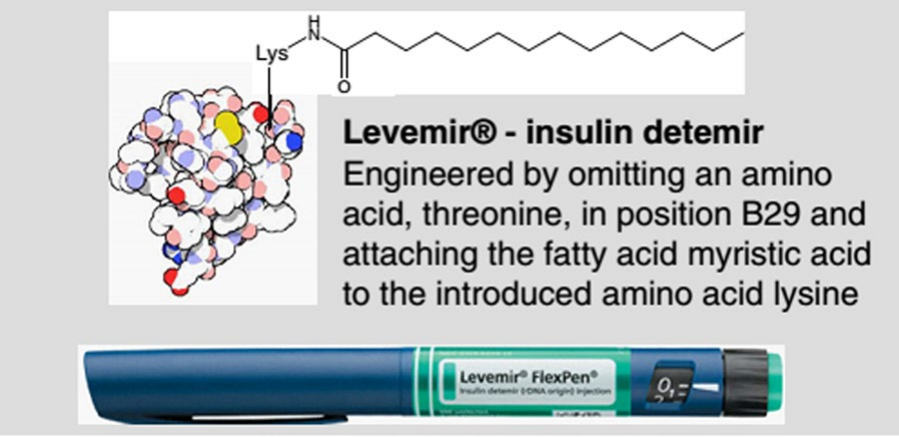
Structure of the drug Levemir used to treat diabetes [199, 200, 228, 229]

**Fig. 21:**
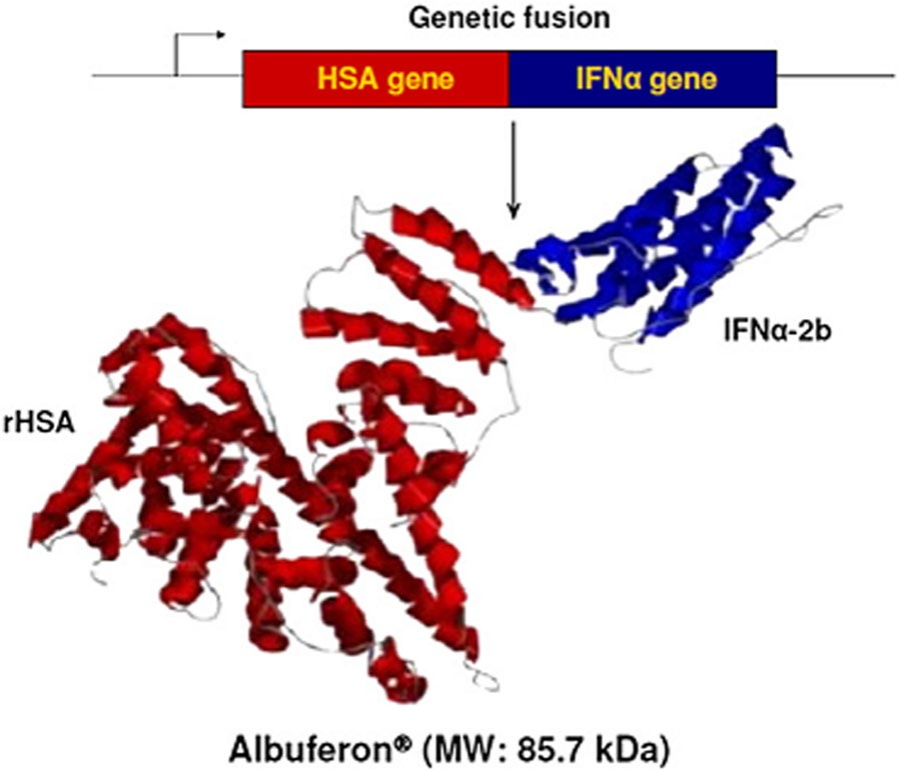
Alboferon is a genetic engineering product derived from the binding of the human albumin gene sequence to interferon alpha [199, 200, 228, 229]

**Fig. 22:**
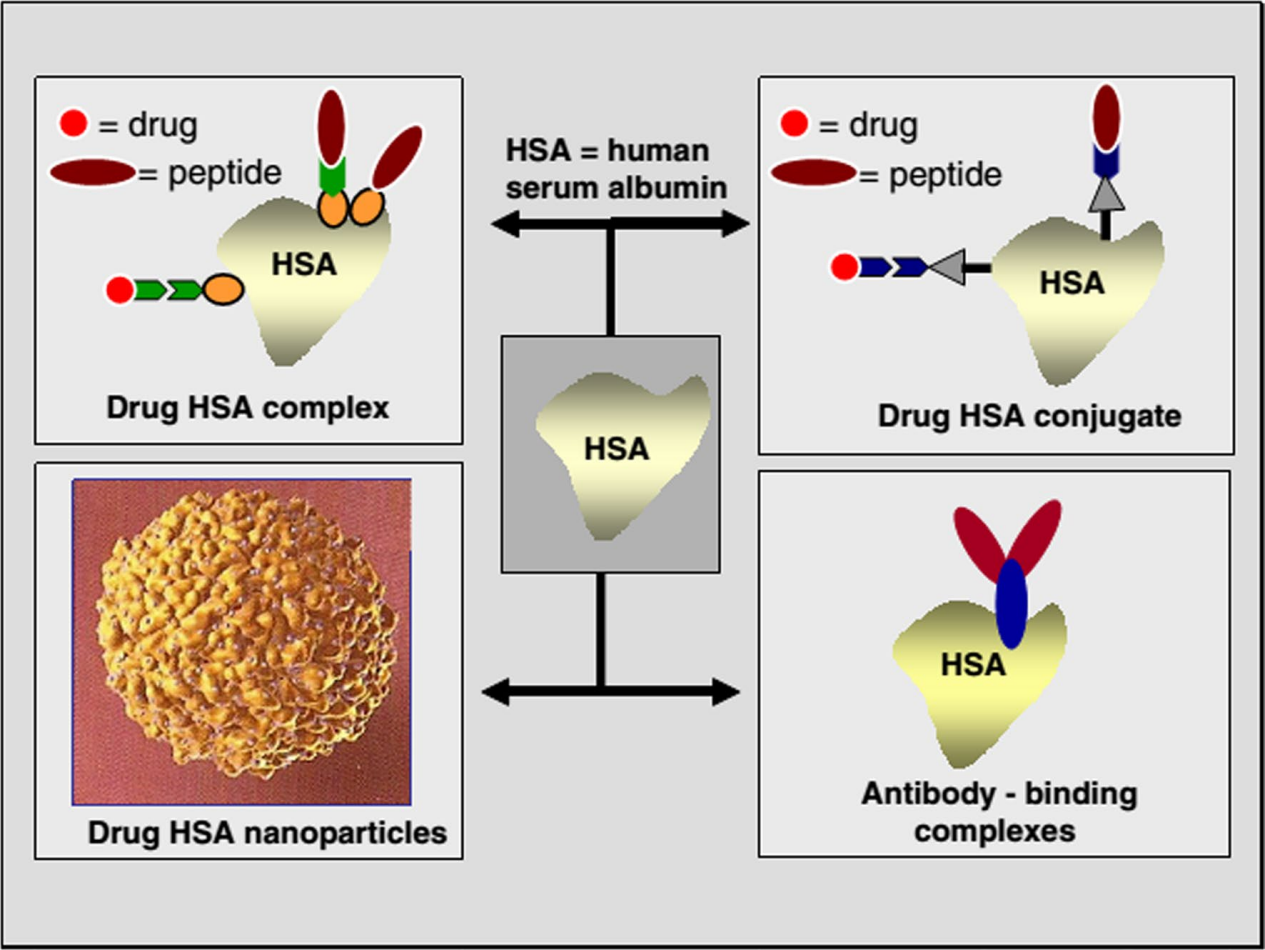
Types of pharmaceutical formulations made using albumin as a carrier [199, 200, 228, 229]

1. Albumin is one of the most important proteins in blood plasma and has many important physiological roles. The presence of high levels of albumin in the body makes the injection of significant amounts of it into the body without side effects or with low side effects. A history of albumin sensitivity is rare in individuals. One of the major advantages of albumin in mass production is its relatively easy access to the source and its price [230, 231].
2. Albumin nanoparticles are biocompatible, non- Taxol, immunogenic and biodegradable, and the residues from their degradation are amino acids that are used as a structural unit to make body proteins by the surrounding tissues.
3. Albumin has many different functional groups and therefore has the capacity to bind to significant amounts of the drug. Full understanding of the amino acid sequence and structure of albumin and multiple charged groups allows the binding of various drugs to albumin nanoparticles by various mechanisms including electrostatic attraction with negatively charged drugs (such as ganciclovir), positively charged ones (such as oligonucleotides), and dual compounds. Gives friend (like doxorubicin) and hydrophobic (like paclitaxel). Also, the presence of multiple functional groups on the surface of the resulting albumin nanoparticles, such as thiol, amine and carboxyl groups, makes it possible to easily change the surface and attach different ligand molecules to its surface to make nanoparticles with different activities and purposes [228, 229].
4. Various albumin-based drug delivery systems are commercially available and available in the market, including levmir and victoza in the treatment of diabetes, ozoralizumab in the treatment of rheumatoid arthritis, albuferon in the treatment of hepatitis C and the product. Albures 99mTc and Tc-Nanocoll 99 m can be mentioned in nuclear medicine (Figs. 20, 21), and albumin is also used as a carrier in the treatment of cancer and viral diseases. The four major methods that use albumin as a drug carrier are shown in Fig. 22. Drug precursors, and proteins and peptides can bind directly to this protein by non-covalent and covalent bonds or indirectly through an intermediate ligand having an albumin-binding group (top right and left). Alternatively, nano bodies are attached to albumin, or albumin replaces part of the immunoglobulin G antibody chain (fragment (Fc (bottom right)). Using albumin nanoparticles another important strategy has been to use albumin as a drug carrier (bottom left).
5. There are different methods for making albumin nanoparticles under mild environmental conditions.
6. Albumin as a structural unit of nanoparticles [232–234], an acidic protein, is very soluble and stable. Albumin is a flexible molecule and easily deforms depending on the environmental conditions in which it is located and also by changing the binding of ligands and returns to its original state at the first opportunity with the help of disulfide bonds, and this property is an advantage. It is important for it in the physiological environment and outside the body. This protein is able to regenerate its structure even though its numerous disulfide bridges are broken. Albumin does not have the conventional properties of most proteins, as it is a very stable and potent protein that, unlike many proteins, is in a wide range (pH = 4–6) and for a long time at high temperatures (more than 10 h at room temperature Above 60 °C) and also remains active in organic solvents. Its denaturation occurs only in non-physiological environments with severe changes in temperature, pH and ionic concentration of the environment. Introduces all the mentioned properties of albumin as a suitable subunit for making nanocarriers in drug delivery [199, 200, 228, 229].
7. As an anti-cancer drug formulation, albumin nanoparticles [235–237] accumulate in tumor tissue both through passive targeting and through active activation targeting, and therefore albumin nanoparticles have a high therapeutic ability. They have malignant solid tumors.
8. In general, albumin nanoparticles allow better control of drug release than liposomal formulations, which is effective in improving patient satisfaction and acceptance.

## Albumin

**Fig. 23:**
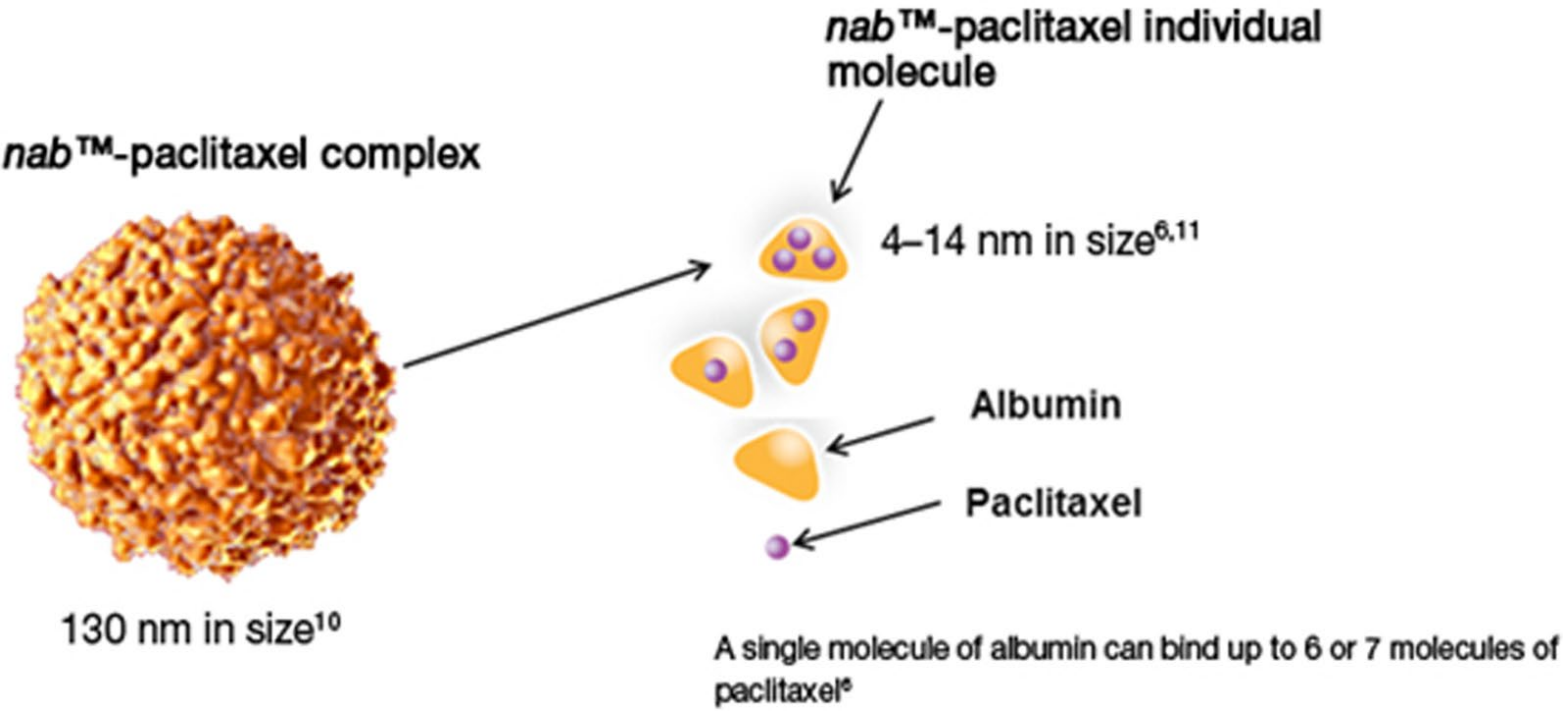
Structure of Abraxane (nabTM-paclitaxel)

Over the past few decades, albumin has emerged as a powerful macromolecular carrier in medical therapeutic and diagnostic applications. This protein with a half-life of about 19 days in the bloodstream can play an important role in improving the pharmacokinetic properties as well as targeting drugs [238]. Abraxane Albumin Formulation Paclitaxel Anti-Cancer Drug Benefits the benefits of albumin in its antitumor function (Fig. 23).

## Types of albumin

For commercial use, albumin is made from a variety of sources, such as egg white called ovalbumin, bovine serum albumin (BSA), and human serum albumin (HSA). Milk, soy and legumes are other sources of albumin [239].

## Ovalbumin

Ovalbumin is one of the most widely used food proteins, which is widely used in the food industry. This molecule is a glycoprotein with a molecular weight of 47 kDa, contains 385 amino acid roots and has only one disulfide bond. The main reason for choosing and using this protein as a drug carrier is benefits such as easy access to its source and its cheap price. Other properties include the ability to form gels, suspensions and foams. Due to its pH-sensitive and temperature-sensitive properties, ovalbumin has the potential to be used as a drug release control agent [228].

## Bovine serum albumin

This protein with a molecular weight of 69 kDa is widely used in drug delivery. The popularity of this protein is due to various advantages such as abundance of its source, cheap price, easy separation and purification from bovine serum, its high capacity to bind to ligands and also its wide acceptance in the pharmaceutical industry [240].

## Human serum albumin

**Fig. 24:**
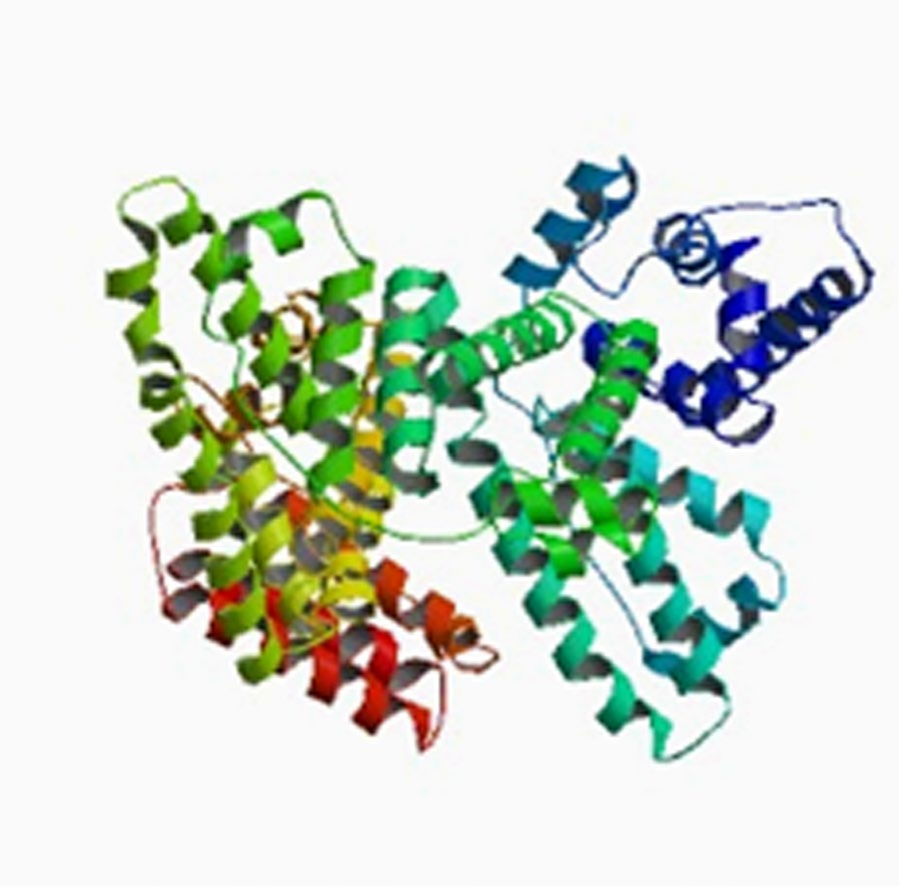
Albumin structure. Albumin is a polypeptide strand containing several alpha chains [199, 241]

**Fig. 25:**
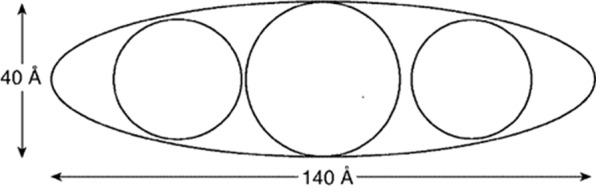
Shows the schematic structure of albumin in solution [199, 241]
- Physiological functions of albumin
  Albumin is one of the most important plasma proteins and has many important physiological roles. This protein makes up more than 60% of the mass of plasma proteins and its amount is about 35–50 g per liter of blood serum. Albumin alone is responsible for more than 80% of the plasma osmolality pressure, and also plays an important role in stabilizing blood pH by its buffering action. This protein, like many plasma proteins, is made in the liver and has a daily production rate of 10–15 g in the body, and its average half-life in human blood serum is 19 days. Albumin acts as a carrier of many molecules including fatty acids, eicosanoids, bile acids, steroid hormones, vitamins C, D, folate, copper, zinc, calcium, magnesium, as well as many drugs in the blood such as penicillin’s, sulfonamides, benzodiazepines And end lytic compounds are involved (Fig. 26). In its protective role, albumin binds to the toxic metabolite bilirubin and transports it to the liver for excretion. Albumin exerts its protective role by binding to toxic substances of external origin such as benzene and the carcinogenic compound Afflation and various other compounds. It is used as a therapeutic agent in human serum albumin in the treatment of various diseases such as shock, burns, albumin deficiency, trauma and cardiopulmonary surgery, acute respiratory problems and blood dialysis. Another important feature is its preferential absorption by inflamed tissues as well as tumor tissues, which is an important advantage for use as a drug carrier [200].

- Albumin structure
  Human serum albumin is a spherical soluble protein with a molecular weight of 66.5 kDa consisting of 585 Amino Acid consisting of a single polypeptide strand. This chain is a set of alpha-helix chains that form three separate second structures (Fig. 24). Albumin contains 35 cysteine roots, which play a fundamental role in the formation of the structure of this protein by forming 17 disulfide bonds. Also, the presence of a large number of charged amino acids such as lysine, arginine, glutamic acid and aspartic acid in its structure plays an important role in the various biological roles of albumin and also in the production of nanoparticles and the binding of various factors to it. The three-dimensional structure of human albumin is determined by X-ray Crystallography, according to which the albumin has a heart-like structure with dimensions of 80 by 30 angstroms [199, 241]. Of course, this structure is somewhat different in solution and all three of them are elliptical (Fig. 25).

**Fig. 26:**
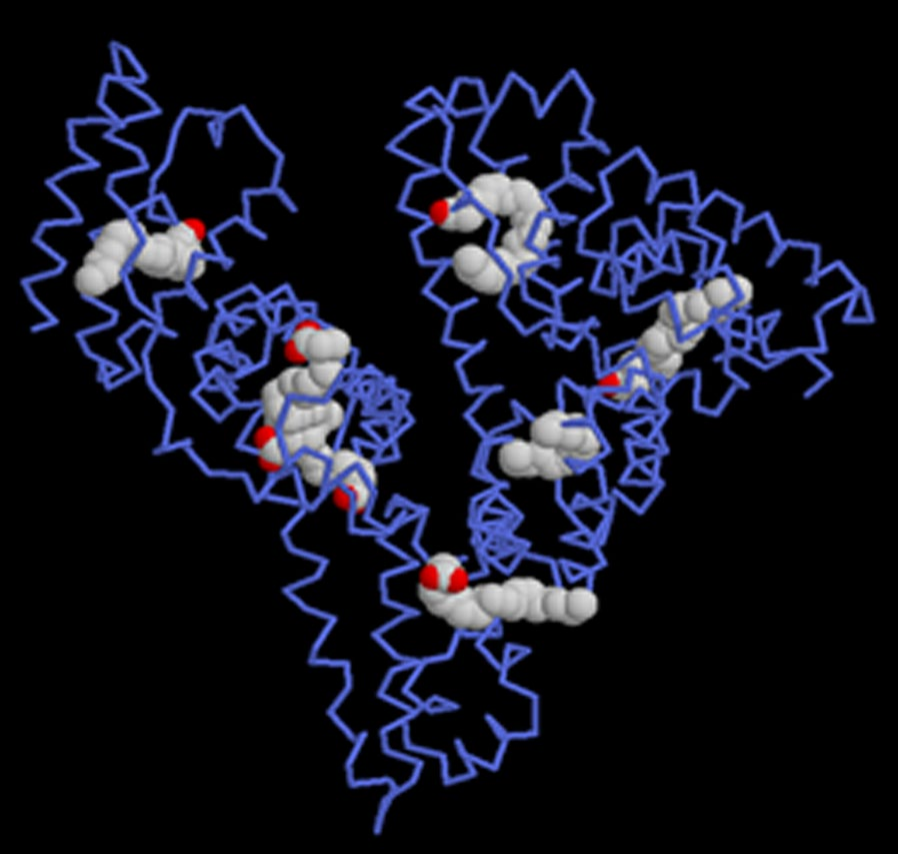
Albumin has multiple binding sites for a variety of biomolecules and drugs [200]

## Mechanism of targeting by albumin nanoparticles

### Passive targeting

**Fig. 27:**
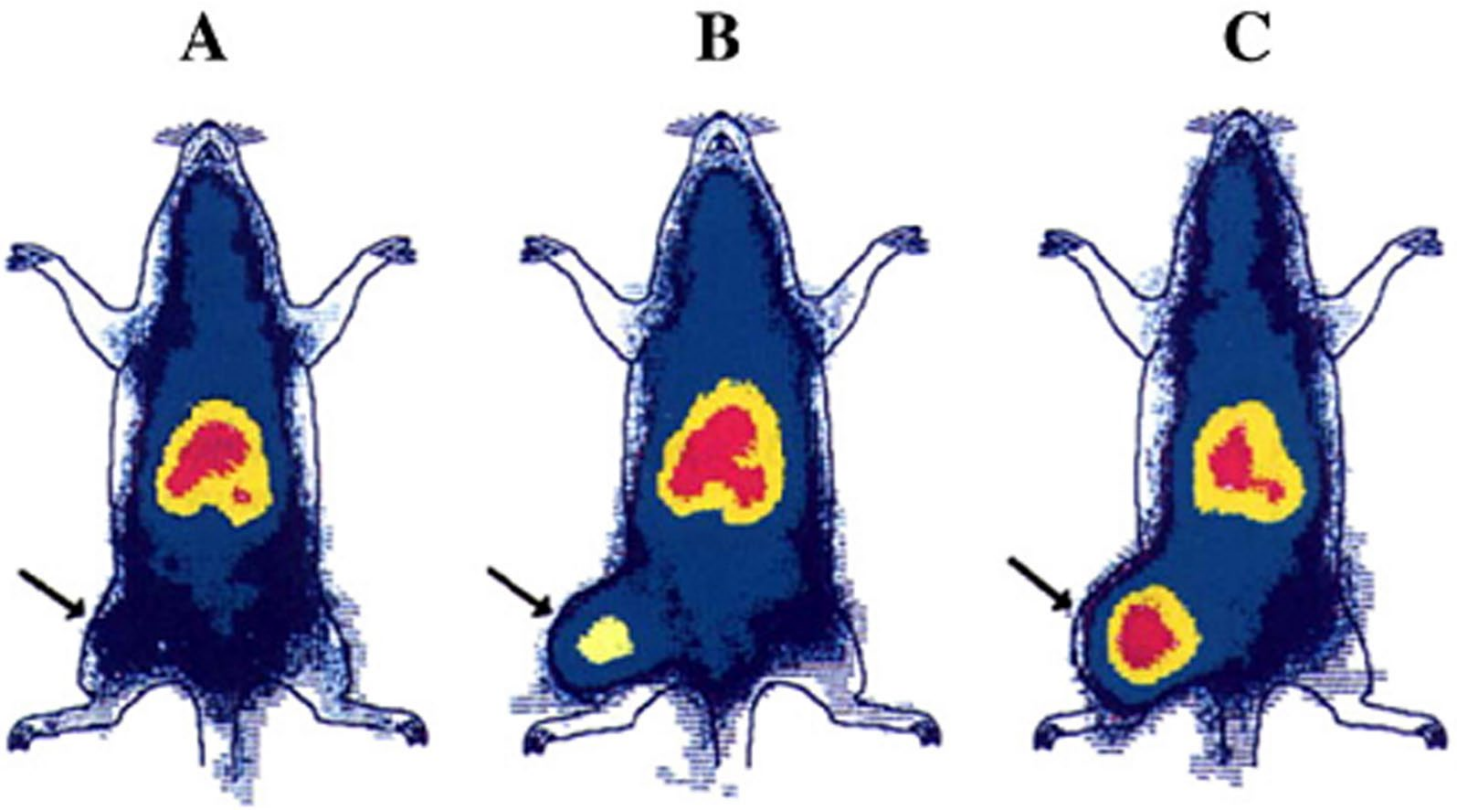
Accumulation of albumin-advance blue complex in a tumor formed in the left leg of a growing rat during (**A**) 24 h (**B**) 48 h, and (**C**) 72 h [199, 200, 228, 229, 238–241]

Tumors have the ability to trap plasma proteins and use their amino acids as a source of energy and food during their proliferation. Tumor vascular endothelial wall is defective and has greater permeability than healthy vasculature, so the entry of some macromolecules into the interstitial fluid space that does not occur in small tissue vessels or occurs in small amounts increases in tumor tissue. On the other hand, lymphatic resection of interstitial fluid in tumor tissue is not performed well and the combination of the two leads to the accumulation of macromolecules in tumor tissue. This phenomenon is called Enhanced permeation and retention. Studies have also shown that the duration Long circulation time is one of the prerequisites for increasing tumor resection of proteins. Albumin with effective hydrodynamic diameter of 7.2 nm and long circulation is a suitable candidate for drug delivery to tumor tissues and EPR mechanism is one of the mechanisms to increase harvesting. It is a tumor (Fig. 27) [199, 200, 228, 229, 238–241].

### Targeted active

**Fig. 28:**
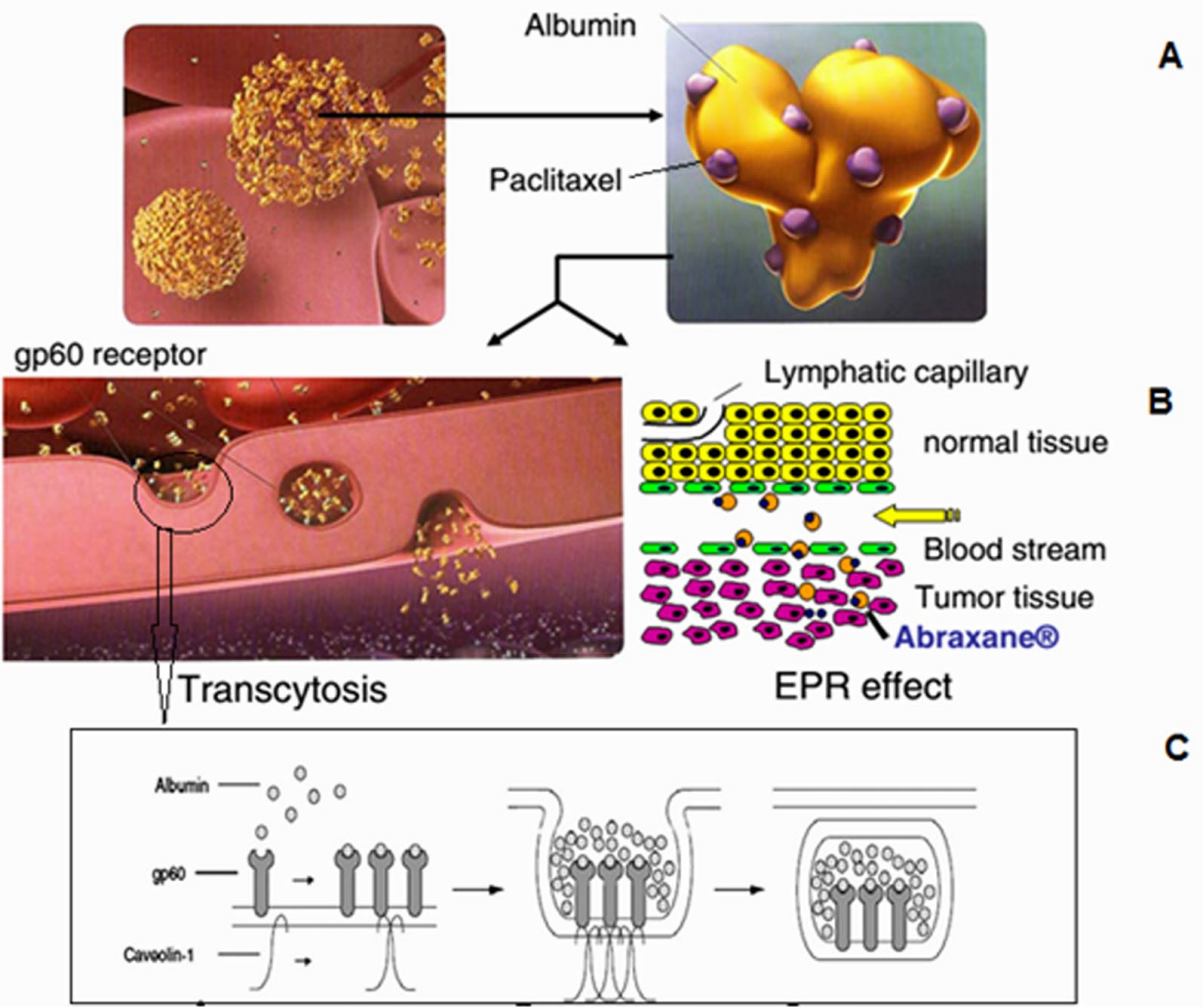
**A** Structure of albumin nanoparticles in Abraham. **B** Two mechanisms that increase the accumulation of abraxas in tumor tissue. **C** Details of the mechanism by which albumin passes through endothelial cells into interstitial fluid space [199, 200, 228, 229, 238–241]

Albumin has a receptor called albobandine or glycoprotein 60 (with a molecular weight of 60 kDa) on the surface of endothelial luminal cells, the drug-albumin complex binds to these receptors. The affinity of the paclitaxel albumin complex for glycoprotein 60 is very high and in the nanomolar concentration range. This binding stimulates the accumulation of glycoprotein 60 molecules and then the accumulation of proteins called kaolin in place. These proteins are involved in the process of endocytosis. Thus, the drug-albumin complex enters from the luminal surface of endothelial cells and on the other hand it is released and enters the interstitial fluid space. In the interstitial space, this complex binds to an extracellular matrix protein called SPARK, increasing the shelf life of drug-containing albumin in the extracellular space and releasing paclitaxel over a long period of time in the vicinity of cancer cells. SPARK protein expression is increased in several cancers, and as a result, this property of albumin nanoparticles leads to active targeted drug delivery to tumor tissue (Fig. 28) [199, 200, 228, 229, 238–241].

### Abraxane commercial formulation of albumin nanoparticles containing paclitaxel

**Fig. 29:**
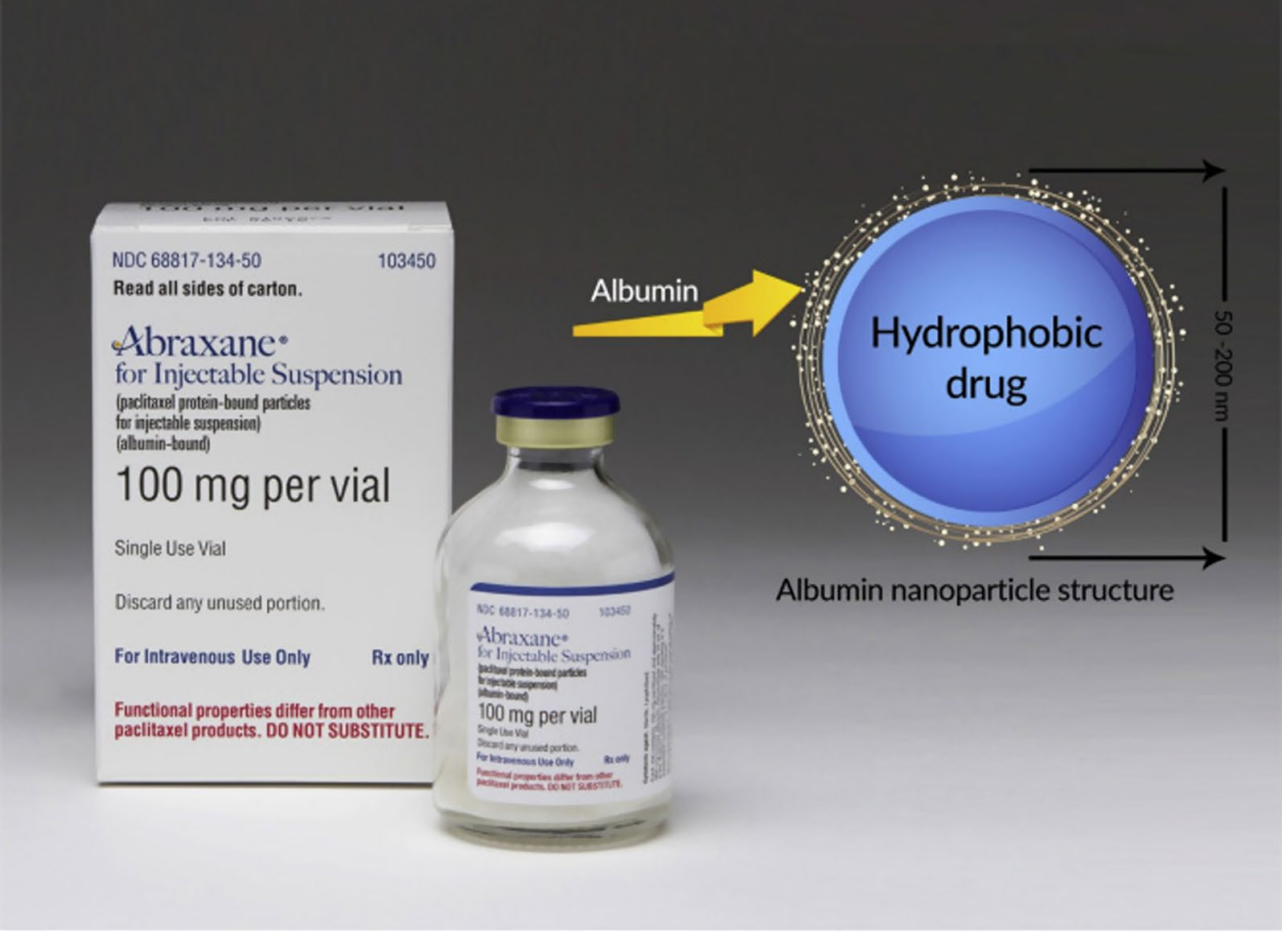
Abraxane formulation containing 135 nm particles of albumin nanoparticles containing paclitaxel [28, 29, 41–51, 55–67, 73–95, 97–159, 165–172, 177–180, 184–202, 228, 229, 238–242]

Taxol are a family of compounds with strong anti-cancer properties, and Paclitaxel is a member of this family. The most important limitation of these compounds for therapeutic use is their low solubility in physiological body fluids. For this reason, Taxol is used as a formulation with higher solubility of Paclitaxel for the treatment of cancer. Abraxane was developed to overcome the limitations of Taxol and has several advantages over Taxol in the treatment of various cancers, which are mentioned below (Fig. 29) [199, 200, 228, 229, 238–241].

**Fig. 30:**
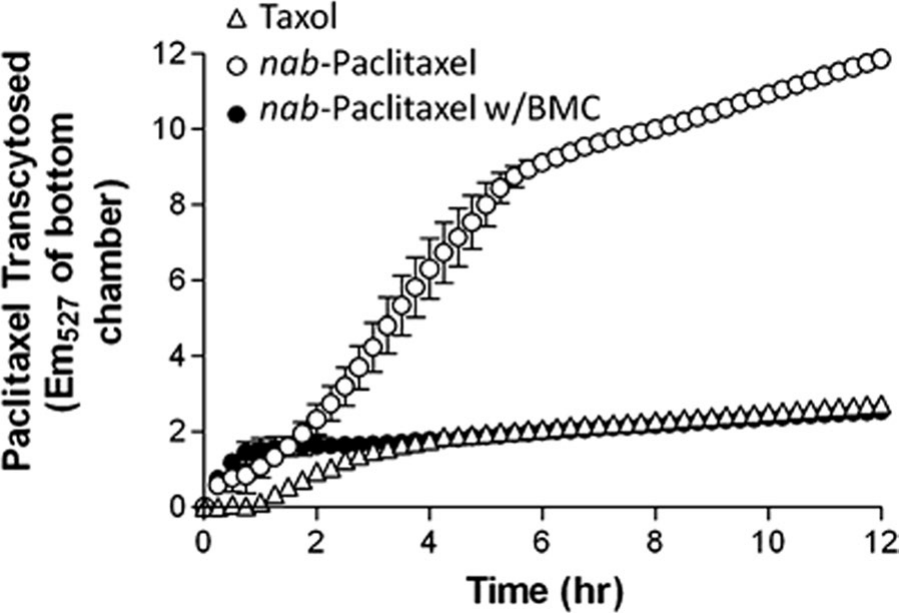
Comparison of paclitaxel entry into endothelial cells in three formulations of Taxol and Abraxane and Abraxane containing methyl beta-cyclodextrin: Abraxane increases the cellular uptake of paclitaxel by 4.4 times compared to Taxol. When cyclodextrin is used as an inhibitor of 60 gp glycoprotein (which, by binding to albumin, causes endocytosis and increases its cellular uptake), the rate of drug uptake is very similar to that of Taxol, indicating a direct role for albumin in increasing paclitaxel cellular uptake [228, 229]

**Fig. 31:**
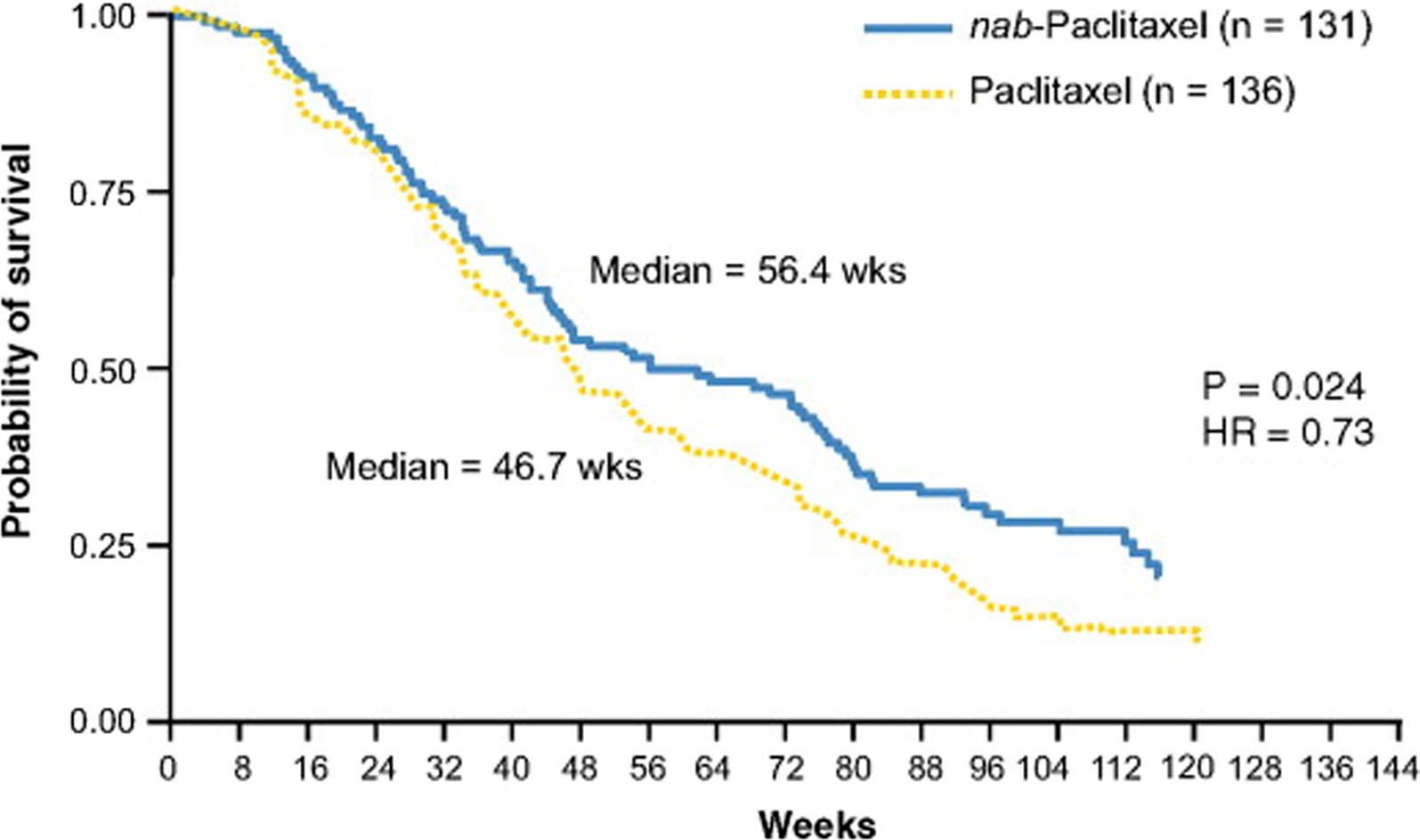
Comparison of the effect of two formulations of Abraxane and Taxol in prolonging the tumor progression process in patients with metastatic breast cancer: Abraxane causes a significant delay in tumor progression compared to Taxol in equal time

1. In terms of CrEL-paclitaxel formulation with the brand name Taxol, it contains 50% ethanol and 50% chromophore to improve the solubility of Paclitaxel. Chromophore is a toxic compound that limits the use of Taxol, while nab-Paclitaxel under the brand name Abraxane (ABI 007) contains a formulation of Paclitaxel containing 3 to 4% albumin [199].
2. In terms of injection time, Taxol requires a long injection time of 3 to 24 h, while the duration of Abraxane injection is only 30 min.
3. Before injecting Taxol, it is necessary to prepare the patient by injecting various drugs such as corticosteroids and antihistamines to reduce the risk of hypersensitivity reactions. Including dexamethasone (12 and 6 h before injection), diphenhydramine (1 h before injection) and cimetidine (30 and 60 min before injection) while Abraxane does not require preparation. Also, unlike Abraxane, Taxol injection requires a special injection set that increases the cost of treatment [228, 229].
4. Due to the protection of paclitaxel in albumin nanoparticles in Abraxane, the maximum allowable dose of paclitaxel in this formulation is 260 mg / m^2^, which is significantly higher than Taxol (175 mg / m^2^). It should be noted that despite the possibility of using a higher dose of paclitaxel to treat cancer in Abraxane, the side effects of the drug, including neutropenia, are less than Taxol. Pharmacokinetically, due to linear release, Abraxane is superior to Taxol with non-linear release [228, 229].
5. Studies with radioactively labeled paclitaxel show that the drug passes more than 4 times the width of endothelial cells in the formulation of Abraxane relative to Taxol (Fig. 30) [199, 200, 228, 229, 238–241].
6. Intracorporeal studies show that at an injectable dose equal to paclitaxel in the two formulations of Taxol and Abraxane, the rate of drug accumulation in the tumor when using Abraxane is 33% higher than that of Taxol and Abraxane also causes a significant delay in tumor growth rate (Fig. 31) and also significantly increases the patient's lifetime (survival)[228, 229].
7. It is possible to actively target Abraxane using changes in their levels with antibodies and peptides [228, 229].

### Methods of producing albumin nanoparticles

**Fig. 32:**
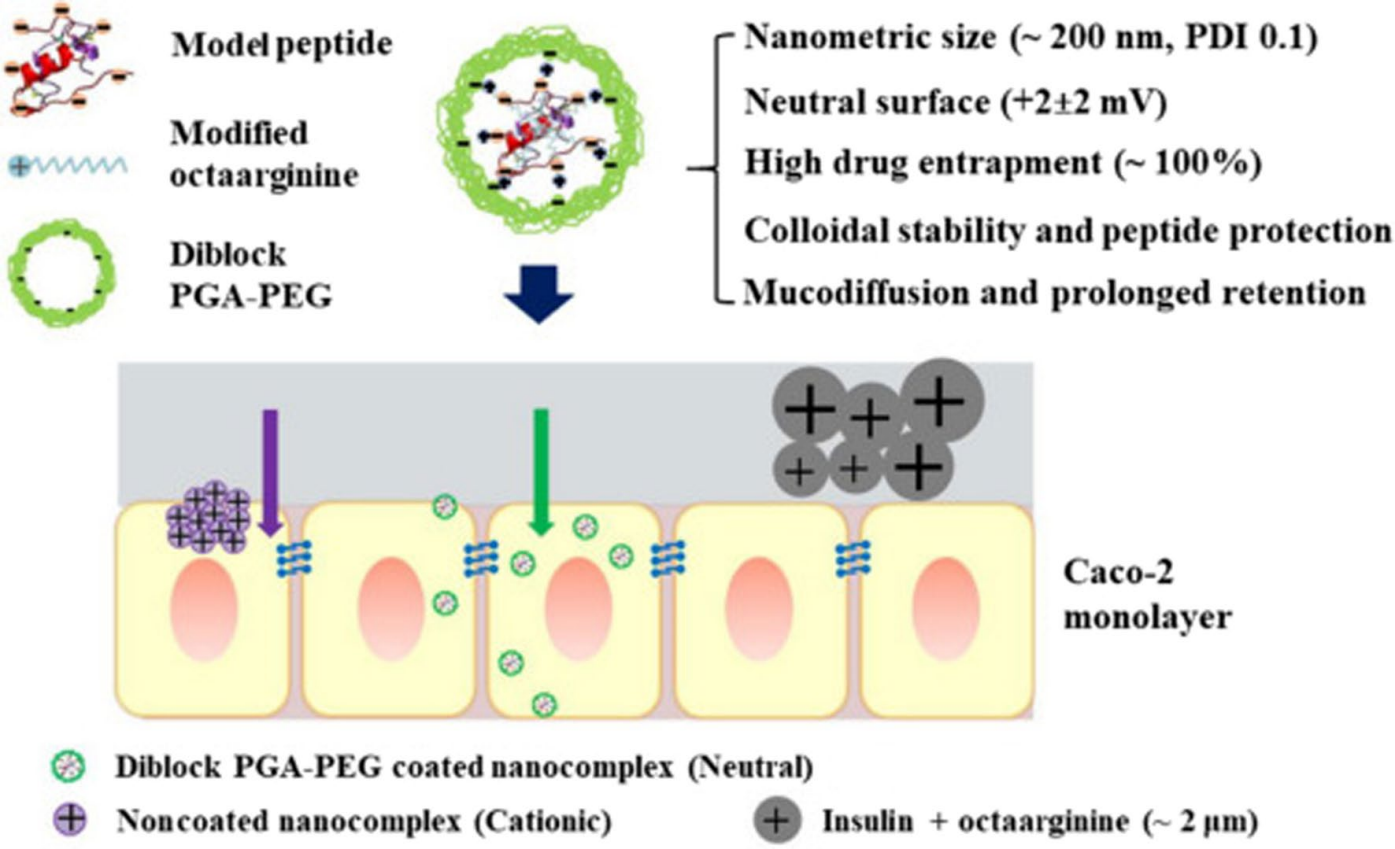
Methods of producing albumin nanoparticles [228]

**Fig. 33:**
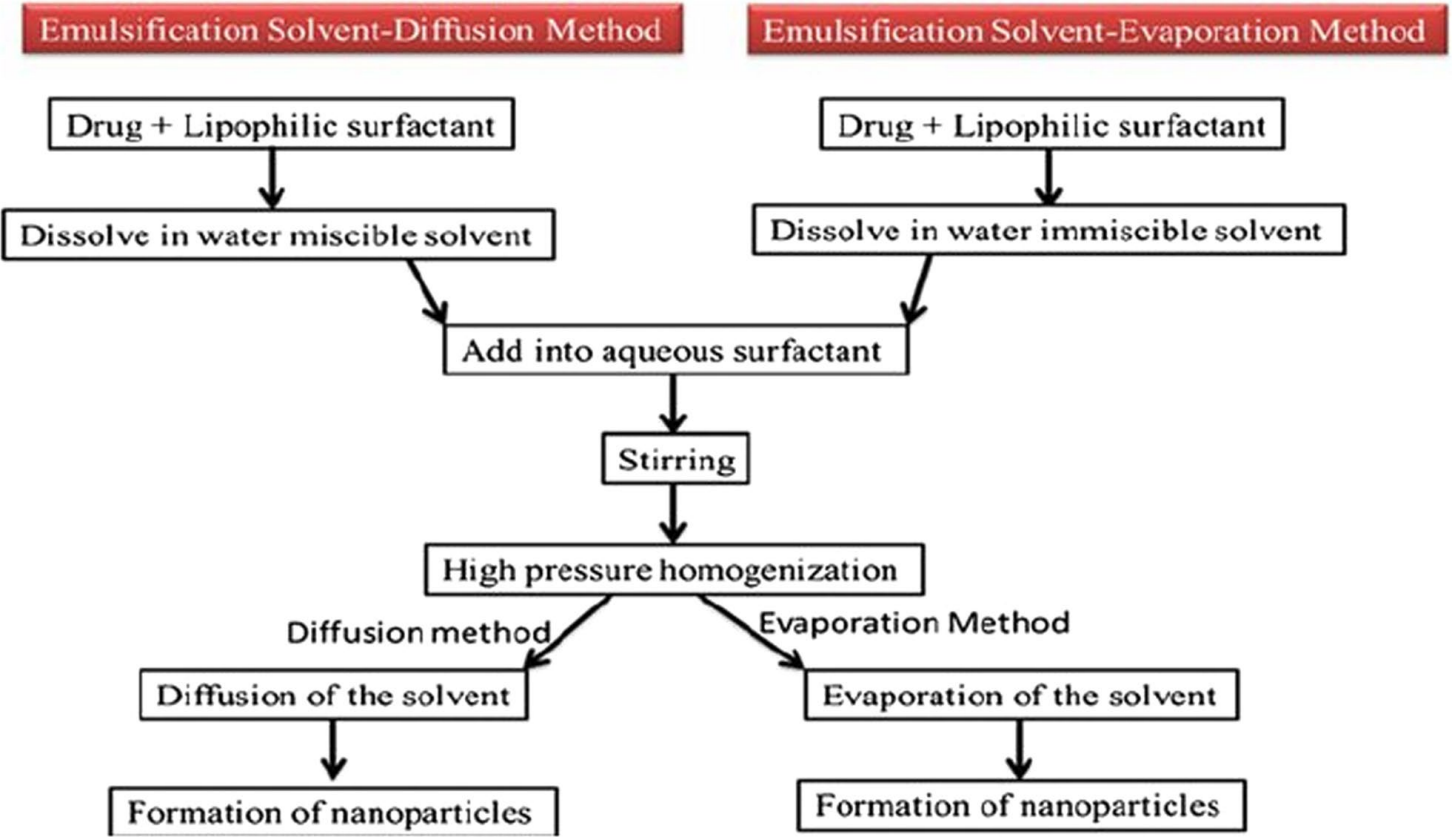
Albumin nanoparticles by emulsion-evaporation method [199, 200, 228, 229]

There are several methods for making albumin nanoparticles [243–245], the most important of which is the emulsion-evaporation method used in the manufacture of Abraxane (Fig. 32). Some of these methods are [199–202, 228, 229, 238–241]:

1. Emulsion method—evaporation with the creation of cross connections (emulsion evaporation cross link method),
2. Phase separation method,
3. Simple coacervation method,
4. Self-assembly method (self-assembly),

5. Nano spray drying method and thermal method (Thermal gelation).
6. Production process of Abraxane formulation (nab-Technology): The basis of nab (nanoparicle albumin bound technology) is the use of evaporation emulsion method with the creation of crosslinks between albumin units for the stability of the resulting nanoparticles. The generalities of its production process are given (Fig. 33).

## Mathematical modelling of drug release kinetic

Equation (1) [246, 247, 248, 249] was used to quantify the normalized drug release from nanocarriers:

1 $$Release=\frac{M_t}{M_f}.$$

The total quantities of drug released at any time (t) and the final amounts of drug released, respectively, are M_t_ and M_f_.

The stabilized drug release primarily for the early stage (0–8 h), which can be represented by Peppa’s model [250], a statistical and semi-empirical model:

2 $$Release=K t^{n},$$

where k is determined by the liposome's and drugs structural properties, and n is determined by the drug's release mechanism (Fickian diffusion or non-Fickian diffusion) and carrier geometry. (Times) is the dimension of k, and n is dimensionless.

In order to align the experimental drug release with models [251–254], nonlinear regression analysis was conducted using MATLAB software version 7.8 (Mathworks Inc., Natick, MA) [255–259].

The consistency of the fit was determined using root mean square error (RMSE) [260–265] and R-square (R^2^) [266–269]. A better fit is shown by higher R^2^ and lower RMSE values [238–239]. Expressions (3) and (4) can be used to quantify these:

3 $$RMSE={\left[{{1/N {\sum_{i=1}^{N}}{{{\left({Release (\%)_{pre, i}} - Release (\%)_{exp,i}\right)}}}}^2}\right]}^{\frac{1}{2}}$$

4 $$R^{2}=1-\frac{\sum_{i=1}^{N}( Release (\%)_{pre, i} - Release (\%)_{exp,i})^2}{\sum_{i=1}^{N} (Release (\%)_{pre, i} - Release (\%)_{exp,i})^2}$$

where release (percent)exp,i is the experimental release used in every calculation, release (percent)pre,i is the expected release for this measurement, N is the number of observations, Z is the number of model constants [270–272], release (percent)exp is the total average data, and I is ith data, and N is the number of observations, Z is the number of model constants, release (percent)exp is the total average data, and release (percent)exp is the total It is assumed that there is no barrier to drug delivery in the buffer solution. Multiple regression methods were used to construct drug release model coefficients with different parameters such as buffer pH and temperature in order to generalize the drug release kinetic model [273, 274].

## Conclusion

1. Nanoparticles are among the most promising carriers in modern drug delivery systems. Among these, protein nanoparticles due to numerous advantages such as easy access to their resources, renewable resources, reasonable price, biocompatibility and biodegradability, the existence of multiple functional groups to carry large amounts of drugs and the possibility of connecting targeting groups to them. To target nanoparticles to a specific target cell or tissue, they are considered. Various animal and plant proteins have been used to make nanodrates. Among the most important animal proteins used to make nanocarriers are gelatin, collagen, and elastin, milk proteins such as casein, albumin, and silk fibroids.
2. Due to their high hydrophobic nature, some plant proteins have the ability to produce nanocarriers that, unlike animal proteins, do not require chemical linkers to produce stable nanoparticles and can also retain their drug shipments for long periods of time. They have a long-term release of drug delivery systems. Abundant resources and easy and cheap access are other benefits of plant protein nanocarriers. Researchers in the pharmaceutical and medical industries are always looking for carriers whose repeatability is high during mass industrial production and it is possible to control their various properties, such as the connection of targeting agents. The properties of protein nanocages promise the production of ideal nanocarriers in the future.
3. Albumin is one of the most important proteins in blood plasma and has had several therapeutic applications in the past few decades. As a rich protein, blood has good biocompatibility and biodegradability. The existence of multiple functional groups makes it possible to carry significant amounts of therapeutic and diagnostic factors as well as targeting factors. It also has a high structural stability and can withstand a wide range of temperature and pH without adversely affecting their structure. The unique properties of albumin have led to the development of a variety of drug formulations based on this protein in the treatment of various diseases. Abraxane nanoparticle formulation the anti-cancer drug paclitaxel is currently used as an effective formulation in the treatment of several common cancers and is in the final stages of clinical trials for other cancers.
4. In the field of nanoparticle drug delivery, protein polymers and protein composite materials are gaining popularity. Their properties are suitable for drug delivery systems, and they have the potential to improve controlled release or targeting processes. Natural protein polymer is an appealing commodity from an economic standpoint because it is comparatively inexpensive, simple to produce, and reusable. Biodegradability and biocompatibility are the key benefits of protein-based nanoparticles over conventional materials. A key factor in deciding the effectiveness of a drug delivery operation is minimizing the host immune response. The aggregation of particle byproducts is reduced by the normal breakdown of these protein polymers, which is also safer for human wellbeing. The properties of protein materials such as silk fibroin, keratin, and elastin, as well as their use in nanoparticle drug delivery and biomedical applications, were the subject of this study. Protein-based nanoparticles can be processed in a number of ways, allowing their properties to be tailored for particular applications. Although there are still obstacles to conquer, there is a growing need in the medical sector for biocompatible protein nanoparticles. To address these obstacles, future research on protein-based nanoparticles must concentrate on the creation of large-scale manufacturing techniques that enable these particles to be produced in a commercially viable manner. To reduce off-target impact, functionalized particles capable of targeting particular areas of the body are likely to be produced. The fabrication and characteristics of protein nanoparticles must change as new pharmaceuticals are developed in order to provide suitable vehicles for drug delivery. The further new experiments are published and the functionality of these protein materials improves, the more people will be interested in them.

## Data Availability

All data generated or analyzed during this study are included in this published article.
